# Chitosan–Oxidized Pullulan Hydrogels Loaded with Essential Clove Oil: Synthesis, Characterization, Antioxidant and Antimicrobial Properties

**DOI:** 10.3390/gels10040227

**Published:** 2024-03-26

**Authors:** Dana Mihaela Suflet, Marieta Constantin, Irina Mihaela Pelin, Irina Popescu, Cristina M. Rimbu, Cristina Elena Horhogea, Gheorghe Fundueanu

**Affiliations:** 1 “Petru Poni” Institute of Macromolecular Chemistry, Grigore Ghica Voda Alley 41A, 700487 Iasi, Romania; dsuflet@icmpp.ro (D.M.S.); impelin@icmpp.ro (I.M.P.); ipopescu@icmpp.ro (I.P.); ghefun@icmpp.ro (G.F.); 2 Faculty of Veterinary Medicine, “Ion Ionescu de la Brad” University of Life Sciences, Mihail Sadoveanu Alley 8, 700489 Iasi, Romania; crimbu@yahoo.com (C.M.R.); rebegeacristina@yahoo.com (C.E.H.)

**Keywords:** chitosan, clove oil, hydrogels, Schiff base covalent cross-linking, biological properties

## Abstract

Emulsion hydrogels are promising materials for encapsulating and stabilizing high amounts of hydrophobic essential oils in hydrophilic matrices. In this work, clove oil-loaded hydrogels (CS/OP-C) are synthesized by combining covalent and physical cross-linking approaches. First, clove oil (CO) was emulsified and stabilized in a chitosan (CS) solution, which was further hardened by Schiff base covalent cross-linking with oxidized pullulan (OP). Second, the hydrogels were subjected to freeze–thaw cycles and, as a result, the clove oil was stabilized in physically cross-linked polymeric walls. Moreover, due to cryogelation, the obtained hydrogels exhibited sponge-like porous interconnected morphology (160–250 µm). By varying the clove oil content in the starting emulsion and the degree of cross-linking, the hydrogels displayed a high water retention capacity (swelling ratios between 1300 and 2000%), excellent elastic properties with fast shape recovery (20 s) after 70% compression, and controlled in vitro clove oil release in simulated skin conditions for 360 h. Furthermore, the prepared clove oil-loaded hydrogels had a strong scavenging activity of 83% and antibacterial and antifungal properties, showing a bacteriostatic effect after 48 and 72 h against *S. aureus* and *E. coli*. Our results recommend the new clove oil-embedded emulsion hydrogels as promising future materials for application as wound dressings.

## 1. Introduction

Essential oils (EOs) extracted from aromatic plants have biological activity due to the content in many molecules with a low molecular weight derived from isoprene (e.g., the terpenoids of related terpenes). It is known that the essential oils are active against Gram-positive and Gram-negative bacteria, possess antioxidant functions, and have been proven to be efficient for skin tissue repair [1,2,3]. However, due to their volatility and instability in different physicochemical conditions (air, light, humidity, and high temperatures), essential oils must be applied carefully, especially in topical formulations. 

The direct incorporation of EOs into hydrophilic matrices is not effective due to their hydrophobicity, and both their antimicrobial effectiveness and their application are limited; higher concentrations being necessary to achieve functionality. In order to maintain their biological activity over a long period, especially for applications in wound healing and tissue engineering, essential oils must be encapsulated in different systems such as oil-in-water emulsions, liposomes, lipidic nanoparticles, or spray-dried microparticles, films, emulsion gels, etc. [4]. Oil-in-water emulsions are formed by dispersing the oil phase into the continuous aqueous phase through emulsification [5]. Polysaccharides (i.e., sodium alginate, chitosan, gum arabic, etc.) [6,7,8], lipids, like soybean oil [9], or surfactants (i.e., Tween, Span, Brij, lecithin, etc.) [10,11,12] are used for the formation of micro- and nanoemulsions. Such systems have demonstrated enhanced loading capacity, stability, controlled delivery, and antioxidant and antimicrobial activities compared to free EOs. Moreover, the emulsion droplets protect the encapsulated active components from oxidation, hydrolysis, and degradation [13]. However, these emulsion systems are thermodynamically unstable due to coalescence, flocculation, and Ostwald ripening processes when a reversion between the two phases occurs. Therefore, improving the stability and efficiency of emulsions is still a challenge.

Emulsion hydrogels (emulsion-filled gels or hydrogel-thickened emulsions) represent a promising and versatile class of materials with applications in various fields, including drug delivery, tissue engineering, and cosmetics. These hydrogels typically consist of oil droplets dispersed in a hydrogel matrix, and they offer unique properties due to the combination of the hydrophobic nature of the emulsion and the hydrophilic nature of the gel. These materials have high stability and bioavailability, requiring lower surfactant amounts when compared with oil-in-water emulsions [14].

The release of the encapsulated substances can be influenced by modifying the composition and the structure of the hydrogel matrix. This inherent property is deeply related to the preparation method. Many reported approaches for hydrogel preparation by emulsification are focused on in situ free radical polymerization (specifically in the emulsion template method) of monomers or prepolymers, which need to use unfriendly initiators [15,16]. To overcome the toxicity-related problems, some authors have used thermally induced polymerization [16] or enzyme-mediated polymerization [17]. Other approaches have used existing polymers, mainly biopolymers, to build the aqueous phase. In this case, the polymeric network is formed by supramolecular self-assembly (i.e., peptide [18]) and ionic and electrostatic interactions [19,20,21,22] or by using other natural compounds as cross-linkers (i.e., phytic acid [23]).

Chitosan (CS), a natural biopolymer derived from the chitin of the exoskeleton of crustaceans, has gained substantial attention in various applications due to its unique properties [24]. CS possesses biocompatibility, biodegradability, and antimicrobial properties, making it suitable for applications in drug delivery, wound dressings, and tissue engineering [25]. It has been proved that CS forms a stable coating layer for emulsions through electrostatic interactions with anionic surfactants [26]. For example, Purwanti et al. [6] showed that emulsions of clove oil (CO) in 1% (*w*/*w*) CS are stable for 29 days of storage; the size of the CO droplets and the physical stability of the emulsions being influenced by the homogenization speed. When incorporated into emulsion hydrogels, CS contributes to the stability and functionality of the system. Thus, when used as a thickening polymer in formulations of *Pelargonium graveolens*, oil-hydrogel-thickened nanoemulsions, CS enhanced the physical stability and antimicrobial properties of the system [27]. Also, CS hydrogels embedding a clove oil nanoemulsion were effective for transdermal delivery of 8-methoxsalen [28]. Hydrogels obtained by the oxidative cross-linking gelation of a quaternary thiol chitosan derivative demonstrated an improved stability for the primary olive oil nanoemulsion and enhanced antimicrobial, anti-biofilm, and antioxidant properties of the oil after incorporation into the hydrogel [29]. 

To increase the mechanical strength and stability of emulsion hydrogels that contain CS, different cross-linkers, such as glutaraldehyde [30,31], epoxy compounds [32], or less toxic genipin [33], can be used to encapsulate and load EOs. Recently, researchers have focused on identifying nontoxic and biocompatible cross-linkers for CS. Notably, the use of dialdehyde polysaccharides as cross-linkers has, as its main result, hydrogels which display unique physicochemical and mechanical properties [34,35]. Pullulan (P), an exopolysaccharide obtained from *Aureobasidium pullulans* fungus by biosynthesis [36], has outstanding biological (biocompatibility, biodegradability, adhesivity, non-immunogenicity, non-toxicity, etc.) and physical (edibility, water solubility, high chain flexibility, etc.) properties [37]. The oxidation of P has been reported by using TEMPO [38] or periodate [39,40], when carboxyl or aldehyde groups are introduced to the main chain. Reactive aldehyde groups could form stable hydrogels by (hemi)acetal bonds [41] or by Schiff base bonds [42,43] when they react with the amino groups in a similar way to glutaraldehyde. 

Clove oil (CO) is one ideal EO for improving healing and reducing infection in wounds, due to its antioxidant activity and free radical scavenging, anti-stress, and antimicrobial properties [44]. However, due to its hydrophobic nature, low water solubility, volatility, and strong aromatic spicy odor, great care must be taken regarding its direct use in foods, cosmetics, or pharmaceutical products [45]. Also, CO is chemically unstable in air, light, and temperature, affecting its activity during storage [46]. Nanoemulsions of CO enhance its stability and solubility [47] and have a noticeable wound-healing capacity by reducing the presence of inflammatory cells at the wound site and increasing cell viability [48] compared to pure clove oil [49]. 

In this work, biocompatible CS cryogels encapsulating CO with a sponge-like morphology and enhanced physicochemical and mechanical properties were obtained. Practically, the stabilized CO–chitosan emulsions were both covalently and physically cross-linked through the Schiff base reaction between the NH_2_ groups of CS and the dialdehyde groups of OP and by employing the freeze–thaw technique, respectively. This approach avoids the use of toxic small molecular cross-linkers and prevents the formation of secondary products. Moreover, the cryogelation technique ensured the synthesis of hydrogels with high porosity, interconnected pores, and physicochemical and mechanical properties close to the skin. The CO-loaded CS cryogels were investigated using FT-IR spectroscopy, scanning electron microscopy (SEM), swelling capacity, and mechanical strength methods. To find the therapeutic potential of these cryogels, the in vitro release kinetics and antimicrobial and antifungal activity, along with antioxidant actions, were assessed and evaluated.

## 2. Results and Discussion

### 2.1. Preparation and Optimization of CS/CO Emulsion

CS/CO emulsions were prepared and the synthesis parameters were optimized in terms of droplet size and time stability. To ensure that the emulsion is stable throughout the whole process of chemical and physical cross-linking, the stability of CS/CO emulsions was evaluated at different storage times, at room temperature, and after ultrasound homogenization (Table 1). Freshly made emulsions had an average droplet diameter of approximately 1000–2000 nm. For low-CO concentrations, no significant effect of the CS concentration on the size of the emulsion droplets was noticed. However, increasing the CO content from 1% to 5 wt.%, as in the CS/CO 4 sample, resulted in higher average diameters of the droplets. The average size of CS/CO 1 ÷ 3 emulsion droplets containing 1 wt.% CO was around 1500 nm after 4 h of storage. The average size of CS/CO 4 emulsions was around 3000 nm for the same storage time. 

**Figure 1:**
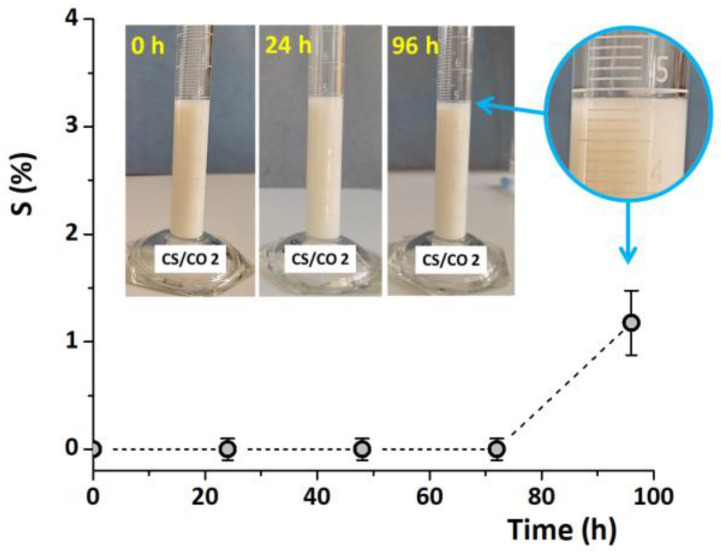
The stability of CS/CO emulsion with 2 wt.% CS and 1 wt.% CO during 4 days of storage at room temperature in the dark.

A slight increase in the size of the emulsion droplets occurred only after 24 h of storage for all CS/CO emulsions (Table 1). Even so, the stability of the emulsions is much higher compared to the reported CO emulsions in 2% acetic acid solutions of CS (1.3%, *w*/*v*), which showed a great instability even after 10–15 min [50]. The better stability of our CS/CO emulsions could be due, on the one hand, to the higher viscosity of the system, attributed to the higher CS concentrations, and, on the other hand, to the final pH of the emulsion (5.5). According to Wang and Heuzey [7], CS can form stable o/w emulsions in the absence of any surfactant or cross-linker because at pH values ≥ 4.5, under ultrasonication treatment, the CS agglomerates are broken and the CS chains adopt a more flexible conformation forming stable emulsions for two months. As shown in Figure 1 the CS/CO emulsions obtained by sonication of the dispersion in fine droplets had a milky aspect. The resistance of CS/CO 2 emulsions against separation, expressed as sedimentation stability (S (%)), indicated that instability occurs after four days of storage, by forming a thin layer at the top of the emulsion (Figure 1), without an observable sedimentation process [51]. This behavior was also noticed by Purwanti et al. [6] for CS/CO emulsions prepared by using 2% Tween 80 after 28 days of storage. This can be explained by the tendency of CO to diffuse from the emulsion droplets into the aqueous phase because of its low-water-solubility property [52]. Following these observations, the hydrogels were further prepared using CS/CO emulsions stored for 2 h at 4 °C, before the start of the destabilization process.

### 2.2. Synthesis and Characterization of CS/OP Hydrogels with and without CO

Hydrogels containing CO with low toxicity, but having high hydrophilicity and improved mechanical properties, were obtained from CS and OP using chemical and physical cross-linking methods. The first method involves the cross-linking of CS with a biocompatible macromolecular cross-linker, oxidized pullulan (OP), while the second concerns the use of the freeze–thaw (F-T) technique. 

It must be underlined that, during the freeze cycles, a large volume of lactic acid solution (herein used as solvent for CS) crystallizes and the ice crystals act as a porogen after their removal by lyophilization. Moreover, since the lactic acid solution forms a continuous phase in the polymer matrix, its removal results in the obtaining of cryogels with interconnected pores. The incorporation of CO into the CS solution by emulsification, before the hydrogel preparation, enabled a uniform distribution of the CO and its better stability in the hydrogel matrix. Usually, the addition of oils or lipids into polysaccharide-based matrices can modify interactions between the chains of polymers; however, the interactions between the polar polymer molecules overcome those between polar polymer and non-polar lipid molecules [53,54]. Therefore, improvement of the intermolecular hydrogen bonds between the free NH_2_ and OH groups of the CS, the OH groups of the OP and CO will be obtained by cryogelation, uniform hydrogel networks with good mechanical properties being obtained.

It is well documented in the literature that hydrogels are easily formed by the reaction of CS with dialdehyde derivatives via the rapid formation of Schiff base bonds [55,56]. Therefore, the oxidized pullulan (OP) with a degree of oxidation of 43%, which corresponds to the 5.26 mmol/g aldehyde groups’ content, and a Mw of 7000 g/mol, was synthesized by using NaIO_4_ as the oxidizing agent [55] and was used both as cross-linker and network polymeric component.

Four samples of CS/OP hydrogels containing CO were prepared (Table 2) by using previously prepared stable CS/CO emulsions (see Table 1). The influence of the cross-linking degree and the CO content of the hydrogels on the properties of the materials were investigated in order to find the best hydrogel formulation for wound dressing applications. 

The entrapment efficiency of CO was between 66.9 and 95.2%, and increased with the increase in the cross-linking degree and oil content (*p*-value < 0.0001), as shown in Table 2. For example, by increasing the oil content from 1 to 5% in the starting CS/CO emulsion, the amount of clove oil entrapped in the hydrogel increased 3.3 times. The increase in the loading efficiency may be attributed to the oil’s hydrophobic nature. Nevertheless, due to the volatile nature of CO, the efficiency may decrease (compared to the theoretical value) because of the vaporization of the oil both during the process of preparing the emulsion by ultrasonication and during the lyophilization of the hydrogel that takes place under an advanced vacuum. In fact, the vaporization of the CO depends on the size of the droplets: the smaller is the size, the higher the surface area of the droplets and, therefore, the faster is the evaporation. As follows, the highest efficiency is obtained when the largest emulsions are incorporated (sample CS/CO 4, Table 2).

The gel fraction (GF) represents a measure of the cross-linking density of polysaccharide hydrogels. Analyzing the GF values (Table 2), the whole cross-linking degree (chemical and physical) of hydrogels increased with the increase in OP content up to 32% (corresponding to a 1:1 molar ratio between CHO and NH_2_ groups, sample CS/OP_1_-C_1_). A further increase in the OP content (48.6%, CS/OP_2_-C_1_) results in a smaller GF value. In fact, high values of OP mean more covalent bonds and therefore a reduced mobility of the macromolecular chains. Therefore, this rigidity of the network may affect the formation of H-bonds (physical cross-linking) during the freeze–thaw process. Obviously, the cross-linking efficiency decreased with the increase in the CO content because the oil obstructs the chemical and physical interaction between macromolecules. 

The hydrogels were characterized by ninhydrin assay to determine the degree of covalent cross-linking. The results showed an increase in the degree of cross-linking by Schiff base bonds with an increase in the molar ratio between the reactive groups of CS and OP (CS/OP_2_-C_1_ > CS/OP_1_-C_1_ > CS/OP_0.5_-C_1_). However, it must be noticed that the covalent cross-linking yield is not high and, the higher it is, the smaller the gel fraction (the overall cross-linking yield decreases). Part of the NH_2_ groups of CS are involved in the stabilization of CO droplets, hence they are not fully available for the cross-linking reaction. 

#### 2.2.1. FT-IR Spectra

**Figure 2:**
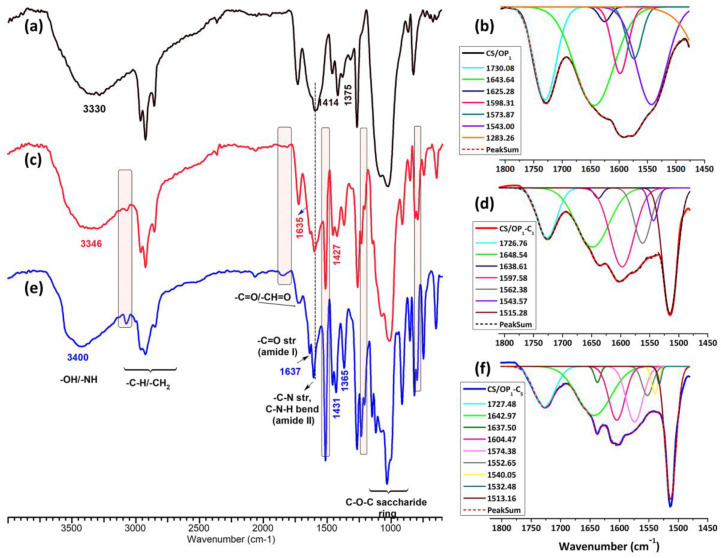
FT-IR spectra and the region between 1500 and 1800 cm^−1^ of the unloaded CS/OP_1_ (**a**,**b**) and the clove oil-loaded CS/OP_1_-C_1_ (**c**,**d**) and CS/OP_1_-C_5_ (**e**,**f**) hydrogels.

The chemical structure of the CO-loaded CS/OP hydrogels was evaluated by ATR-FTIR spectrometry (Figure 2). According to the GC-MSD/FID gas chromatography analysis reported by Suflet et al. [57], 84% of the identified compounds in the CO used in the present study were formed by eugenol (83.11%) and eugenol acetate (0.77%). The second class of components was formed by β-caryophyllene (12.42%), α-caryophyllene (1.19%) and caryophyllene oxide (0.82%), p-allylphenol (0.13%), and L-limonene (0.01%). The other usual compounds, such as esters, alcohols, ketones, and aldehydes, were present in very low percentages (1.55%). The FTIR spectrum of the CO (Figure S1) showed the hydroxyl O–H stretching vibration at 3100 and 3700 cm^−1^, C–H aliphatic peaks in β-caryophyllene in the region 3000–2840 cm^−1^ [58], the carbonyl ester band attached to the aromatic ring of eugenol at 1765 cm^−1^, the C=C stretching vibration of the allyl group in eugenol and allylphenol at 1637 cm^−1^ and 995 cm^−1^, and the C=C stretching vibration of the aromatic moiety at 1607 and 1512 cm^−1^ [59]. The bands at 1431, 1267, 1202–1034, 912, 851, and 795 cm^−1^ belong to eugenol acetate and eugenol and can be attributed to the CH_2_ deformation vibration, the stretching vibration of C–O in the OH-bound C, the stretching vibration of C–C, the C–O–C in ether and alcohol functional groups, C–H rocking –CH_2_–OCH_3_, ring deformation, and the bending vibration of CH_2_ and –CH [60]. 

The FTIR spectra of the CS/OP_1_-C_1_ and CS/OP_1_-C_5_ hydrogels (Figure 2) show the characteristic bands of the starting polymers (Figures S2 and S3), but also an increase in intensity of some bands due to both the presence of the CO and cross-linking bonds by Schiff base (imine bonds) in comparison with the CS/OP_1_ hydrogel. Thus, in the CS/OP_1_ spectrum (Figure 2a), the large band at 3352 cm^−1^ was assigned to O–H and N–H stretching, as well as the intra- and inter-molecular hydrogen bonds; the bands at 2963–2854 cm^−1^ to C–H asymmetric and symmetric stretching from CH_2_ and the CH groups; and the peaks from 1643 cm^−1^, 1591 cm^−1^, 1543 cm^−1^, and 1313 cm^−1^ were attributed to the C=O stretching of amide I, amines, and secondary amides, and C–N stretching (amide III), respectively [61]. The peaks at 1728 cm^−1^ and 1234 cm^−1^ are related either with the lactic acid present in the hydrogel network [62] or with residual –CH=O groups of the OP [40]. At 1456, 1414, and 1375 cm^−1^ the asymmetric and symmetric vibrations of –COO^−^ groups along with O–H bending in the COO^−^ anion are present. The bands of C–O–C of the saccharide ring and polysaccharide structures are in the 900 and 1200 cm^−1^ interval [63]. The intensity of the aldehyde groups band present in the OP spectrum at 1730 cm^−1^ (Figure S3) diminished and a new peak at 1625 cm^−1^ [64], attributed to the imine bonds formed by Schiff- base reaction, was evidenced by the Gaussian deconvolution of the region between 1900 and 1500 cm^−1^ (Figure 2b). In the CO-loaded CS/OP hydrogels (Figure 2c,e), the shifting of the broadband at 3300 cm^−1^ toward higher wavenumber (3346 and 3400 cm^−1^) is assigned to the establishing of H bonds between CO components (eugenol) and OH/NH groups of CS/OP matrix. The intensification and the shift of stretching vibrations of C–O–C bonds in the polysaccharide structure and skeletal stretching vibrations of C–O, respectively, from 1076 to 1070 cm^−1^ and 1018 to 1014 cm^−1^, respectively, confirm the interaction of the CO (its components) with the polymer matrix. The sharp peaks from 3074, 1514, 1431, 1263, 914, and 798 cm^−1^ were attributed to the presence of CO in the sample [57]; a shift of all these bands towards lower wavenumbers was noticed compared with the CO spectrum (Figure S1). Also, in the higher loading sample (CS/OP_1_-C_5_) (Figure 2e), the sharp peaks belonging to CO were more evident and their intensity increased. 

In the deconvoluted spectra of the two CO-loaded samples (Figure 2d,f), the characteristic peaks corresponding to amide I (1643 cm^−1^) and to the amide II (1543 cm^−1^) bonds (un-loaded CS/OP_1_ hydrogel) shifted to 1648 cm^−1^ and 1550 cm^−1^. Also, the absorption bands at 1597 cm^−1^ were weakened with the increase in the cross-linking degree (CS/OP_1_-C_1_ > CS/OP_1_-C_5_, see Table 2), whereas the new band at 1638 cm^−1^ developed and strengthened, this suggests the formation of a Schiff base [65]. 

#### 2.2.2. Morphology and Porosity

**Figure 3:**
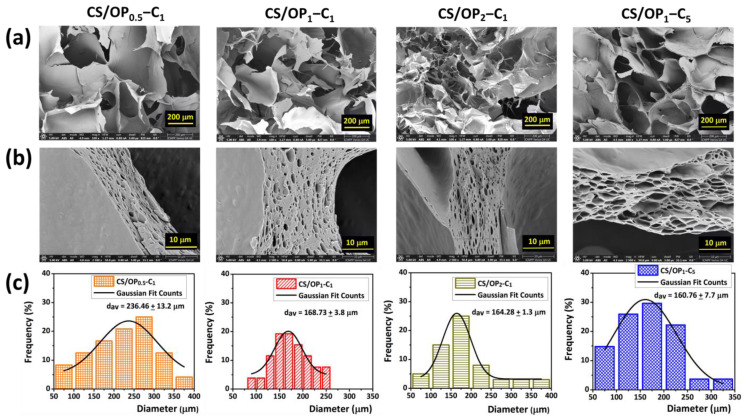
Scanning electron micrographs (cross-sections) of CO-loaded CS/OP hydrogels: general view (**a**), wall detail view (**b**), and their corresponding pore size distribution diagrams (**c**).

As a result of ice crystal formation, a three-dimensional network with irregular geometry and varying macropore sizes (pore diameters in the range of 50 and 380 µm) was observed in the cross-sectional scanning electron micrographs of the hydrogels (Figure 3a). 

As expected, the porous structure and morphology of the hydrogels were influenced by the ratios between the reacting groups. The higher ratio of OP in the CS/OP_1_-C_1_ hydrogel resulted in smaller average pore sizes (168 µm) and thicker walls (~10 µm) compared to CS/OP_0.5_-C_1_ (240 µm and 8 µm, Figure 3a,c). A further increase in the OP content from 32.1 to 48.6% in the CS/OP_2_-C_1_ sample resulted in an augmentation of wall thickness to about 14–15 µm without significant changes in the pore sizes (~164 μm). As shown in Table 2, this sample presents the best covalent cross-linking yield (35.9%) but a smaller GF% (63.9%), since physical cross-linking by H-bonds is obstructed, as previously explained. It must be noted that the CS/OP_1_-C_5_ hydrogel displayed a porous structure, and the increase in the wall thickness to 20 µm and the decrease in the pore sizes to 160 µm suggests a high cross-linked network realized mainly by physical interactions during the freeze drying process.

The images of the cross-section of the hydrogel walls at a higher magnification (Figure 3b) show that the CO had been effectively dispersed and loaded into the hydrogel. The absence of oil droplets in the holes in the cross-section images is due to their vaporization during the SEM examination process, which takes place in a vacuum. The hydrogel sample CS/OP_1_-C_1_ was the best polymeric material because of the effective double degree of cross-linking (covalent and physical) displaying an appropriate average pore size.

**Figure 4:**
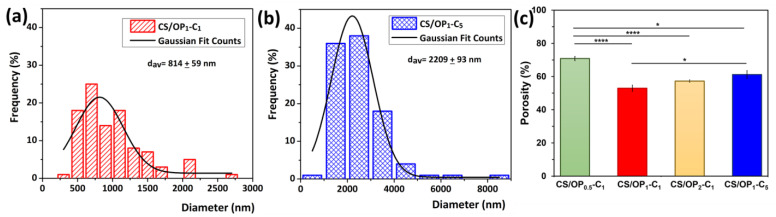
Size distribution diagrams of CO droplets inside the hydrogel walls measured from SEM images (**a**,**b**), and porosity of CO-loaded CS/OP hydrogels measured by the alcohol displacement method (**c**). Results are expressed as means ± standard deviation (S.D.) of three (n = 3) experiments.; * *p* < 0.05; **** *p* < 0.0001, between samples.

The size distribution of CO droplets inside the hydrogel walls measured from the SEM images, compared to hydrogel samples with 1% and 5% incorporated CO (Figure 4a,b), was close to that of the starting emulsion (Table 1), demonstrating that the emulsion was sufficiently stable during the preparation process. Therefore, the average size of the CO droplets in the dried hydrogels increased from 814 ± 59 nm for CS/OP_1_-C_1_ to 2209 ± 93 nm for CS/OP_1_-C_5_. The porosity values of all of the samples measured by the alcohol displacement method (Figure 4c) are in agreement with the SEM data.

Thus, the average porosity decreased from 70.8% to 52.9% with the increase in the OP content from 19.2% to 32.1%; then, with a further increase in the OP content from 32.1 to 48.6%, the porosity slightly increased to 57.2%. This behavior could be attributed to the variation in the cross-linking density of the hydrogels due to the increasing OP content. As seen from Table 2, when the OP content was 32.1%, the theoretical ratio between the reacting groups was close to 1 and the degree of cross-linking (covalent by imine groups and physical by H-bonds) increased. A further increase in the OP content resulted in an excess of CHO groups, which led to a higher degree of covalent cross-linking. However, in this case, the GF% value decreased, resulting in a less-cross-linked network and a more porous structure, as evidenced also by the SEM analysis. The porosity slightly increased from ~52% to ~61% when increasing the CO content of the cryogels (CS/OP_1_-C_5_). The higher concentration and the larger size of the CO droplets may diminish the appearance of large solvent crystals during cryogelation, which leads to the low porosity of the cryogels [66].

The appropriate dimensions of the pores, as well as their high porosity, could be exploited in wound healing for the absorption of exudates. Moreover, the air permeability provided by the interconnected pores enables cell proliferation and re-epithelialization of the new tissue [67]. Similar observations were reported by Bolgen et al., who incorporated *Hypericum perforatum* oil in CS cryogels [66].

#### 2.2.3. Swelling Behavior

**Figure 5:**
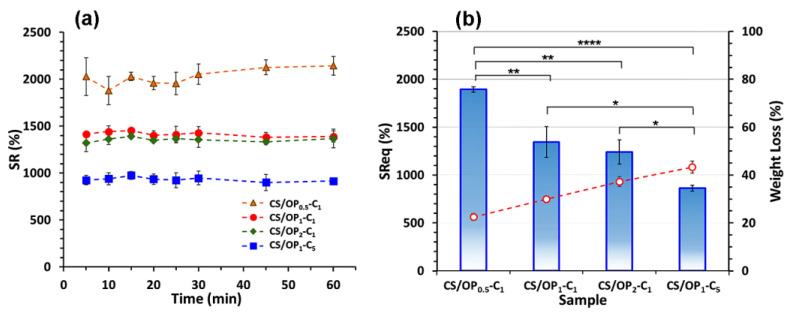
(**a**) Swelling kinetics and (**b**) swelling ratio and weight loss at equilibrium (at 24 h) of CO-loaded CS/OP hydrogels. PBS solutions with pH 5.5 were used in all of the experiments (at 32 °C) and each point represents the average of the results from three different samples (n = 3). * *p* < 0.05, ** *p* < 0.01, **** *p* < 0.0001, between samples.

Using the physiological conditions of simulated skin (pH 5.5 and 32 °C), the swelling behavior of the CO-loaded hydrogels was evaluated (Figure 5a). In these conditions, the swelling is governed both by electrostatic repulsion between the positively charged groups of the CS and by the hydrophobicity of the incorporated CO. The ability of hydrogels to absorb water can be influenced by the dissociation of hydrogen bonds and the relaxation of the polymer chains. Due to the macroporous network structure, the swelling equilibrium of the CO-loaded hydrogels was attained in 10 min. The *SR* values decreased from 2000% to 1400% and then to 1300% as the OP content increased. The increase in the cross-linking degree leads to the decrease in the number of free -NH_2_, -OH functional groups able to interact with the water molecules. Therefore, the affinity of hydrogels towards water molecules decreases in the order CS/OP_0.5_-C_1_ > CS/OP_1_-C_1_ > CS/OP_2_-C_1_. On the other hand, the *SR* values were highly influenced (*p* < 0.0001) by the amount of oil entrapped in the network. Thus, the *SR* value decreased from 1500% to 900% as the CO content increased from 1 to 5% (Figure 5a). In addition to their natural hydrophobicity, the oil droplets occupied the pores in the network walls, resulting in fewer hydrogen bonds between the polymers (CS and OP) and the water, which therefore reduced the water retention capacity (sample CS/OP_1_-C_5_). No significant differences between the *SR*eq values for CS/OP_1_-C_1_ and CS/OP_2_-C_1_ after 24 h were observed (Figure 5b), which indicates the release of non-cross-linked polymers and CO from the hydrogels during the swelling test. These results are sustained by the weight loss of the hydrogel at the end of the experiment. A molar ratio of 1:1 of the NH_2_:CHO groups in the hydrogels revealed a highly cross-linked network (the highest gel fraction of 75.5%) (Table 2). Consequently, the hydrophilicity and direct sorption of water molecules are reduced. The larger amount of CO droplets entrapped in the walls of the CS/OP_1_-C_5_ sample resulted in higher weight loss during the swelling test.

#### 2.2.4. Mechanical Properties

**Figure 6:**
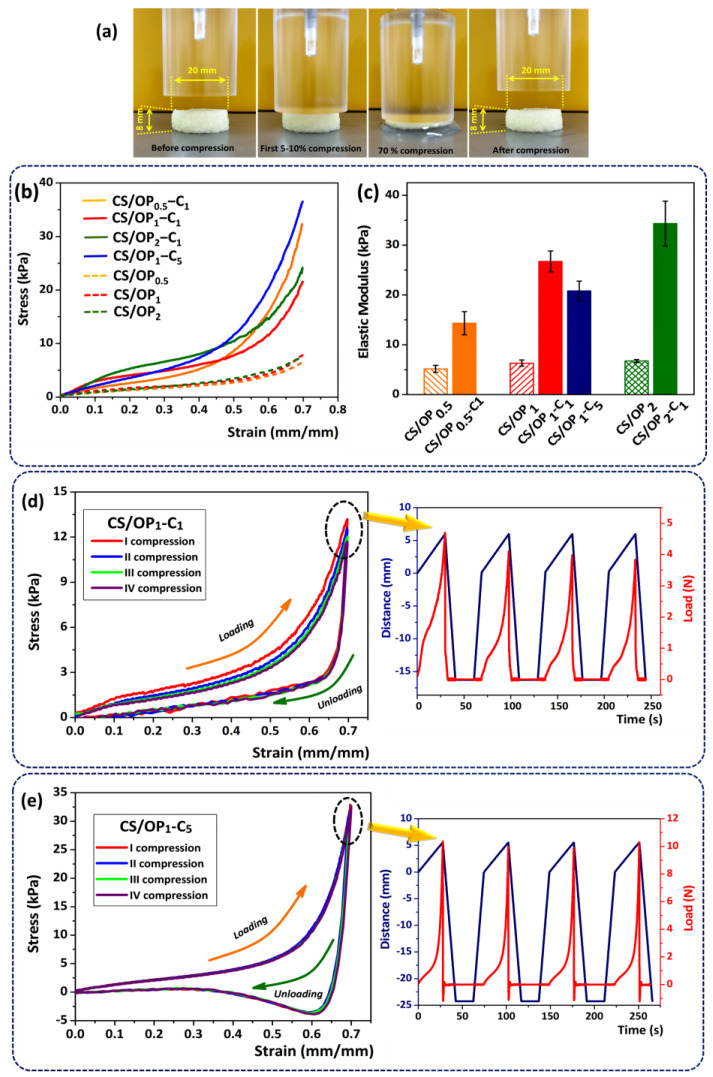
Photographs of the recovery profile of the CS_1_OP_1_C_1_ hydrogel (**a**); Stress–strain curves for unloaded and CO-loaded CS/OP hydrogels tested in a swollen state (**b**); Young’s modulus of all hydrogels was calculated from the slope between 2 and 10% of the linear part of stress-strain curves; (**c**); hysteresis curves for the CS/OP_1_-C_1_ (**d**) and CS/OP_1_-C_5_ (**e**) samples from uniaxial compression cycles recorded at 70% compression.

The compressive strength of the hydrogels represents a mechanical property required for targeted applications. Figure 6 shows the compressive behavior of unloaded and CO-loaded CS/OP hydrogels and their main characteristics. As shown in Figure 6a, all samples subjected to 70% compression in their wet state resisted without breaking and returned to their original shape when the load was released, which is suggestive of their good compressive resistance and shape-recovery properties. 

The representative strain–stress curves (Figure 6b) of CO-loaded hydrogels showed a trend usually found for polymeric foams [68], in which three distinct regions can be observed. The first region is the elastic region and corresponds to the elastic response of the pore walls. Then, a plateau region, characterized by an almost constant or slight increase of the stress with the increase in strain, was observed. This behavior is due to the deformation and collapsing of the pore matrix when water from the cryogel is forced out of the pores. This region ends at the densification point when the stress–strain curve increases due to the fact that compression has completely squeezed the pore network. These typical curves for polymeric foams were also reported for cryogels obtained from gelatin [69] or silk fibroin [70]. 

The most important result was that the mechanical properties of the CO-loaded cryogels significantly exceeded the unloaded ones, with a 2.8- to 5.7-fold increase in their stiffness (Figure 6c). The elastic properties of the swollen CO-loaded CS/OP hydrogels depend on the degree of cross-linking and the CO content. The Young’s modulus values, calculated from the slope of the linear part of the stress–strain curves (Figure 6c), increased from 14.31 kPa (CS/OP_0.5_-C_1_) to 26.74 kPa (CS/OP_1_-C_1_) and 34.31 kPa (CS/OP_2_-C_1_) with the increase in OP amount (increasing the amount of CHO groups). In fact, the hydrogels become slightly stiffer due to the increase in covalent cross-linking points through the Schiff base bonds (as already shown by the *CD* values in Table 2). 

It was observed that the high content of CO in the CS/OP_1_-C_5_ sample (in comparison with CS/OP_1_-C_1_) induces an increase in the elasticity of the hydrogels. This behavior is probably due to the hydrophobic interactions between the oil and the hydrophilic network of the hydrogels and was evidenced by a decrease in the elastic modulus from 26.74 kPa to 20.78 kPa. This is in accordance with the porosity and gel fraction data, when the cross-linking density is reduced with an increase in the amount of entrapped essential oil.

Cyclic compression tests were studied to investigate the recovery capacity of CO-loaded CS/OP hydrogels. Figure 6d,e show the compression test with four consecutive loading–unloading cycles of the hydrogels and with a recovery time of 20 s between compressions. According to the literature, the closed loading–unloading curve of hydrogels with a pronounced hysteresis loop reveals that the hydrogel could recover to its initial state after compression by energy dissipation [71]. Furthermore, there was no obvious shifting and breaking after four cycles at a strain of 70%, which indicated that the hydrogels have good recoverability [72]. In the loading–unloading curves of the CS/OP_1_-C_1_ hydrogel, the highest compressive strength was obtained in the first loading cycle. This behavior can be attributed to the water in the hydrogel, which after the first compression could not rehydrate the hydrogel structure fast enough before the second loading cycle and, therefore, resulted in poorer mechanical properties for the following cycles. Thus, a slight shift of the four hysteresis loops was observed and there was a slight decrease in the load from 4.2 N (cycle I) to 3.73 N (cycle IV) (Figure 6d) [73]. This behavior was not observed in the case of the CS/OP_1_-C_5_ sample when a total recovery (99.33%) and a good overlap of the four hysteresis loops were registered (Figure 6e). The compressive load (N) remained constant throughout the test (10.12 ± 0.19 N) (see detail Figure 6e). This comportment could be attributed to the larger energy dissipation in the CS/OP_1_-C_5_ network, which contains more ductile components (oil droplets) and fewer dense polymeric structures (as shown in Table 2). The sliding friction between CO droplets and the plastic deformation of oil droplets could effectively disperse the applied stress and dissipate energy, providing the hydrogel with greater elasticity and toughness [70]. In addition, the hysteresis curves of the CS/OP_1_-C_5_ hydrogel recorded a decrease below the 0 value of the force (detail Figure 6e), indicating an adhesion to the stainless-steel surface of the device. This behavior may be due to the hydrophobic forces that occur at the interface between the CO and the stainless plane. 

### 2.3. In Vitro Release Studies

**Figure 7:**
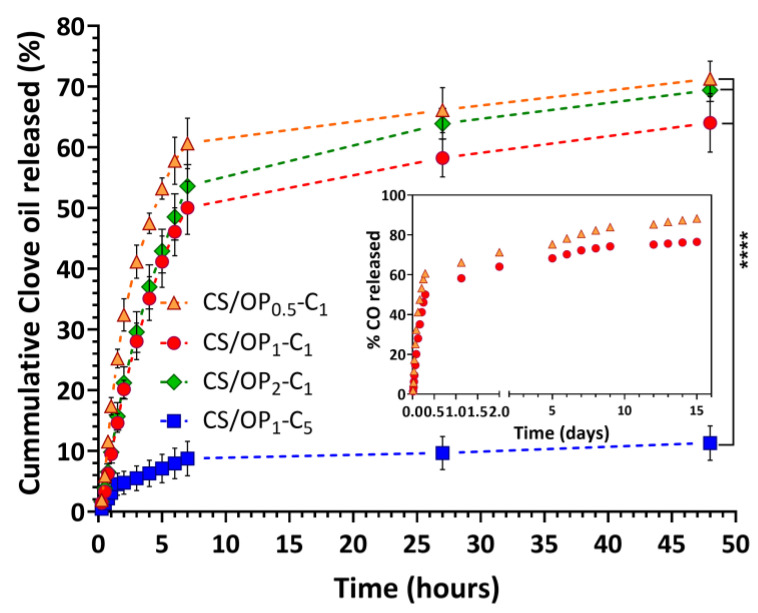
Release profiles of clove oil from the CS/OP hydrogels in a phosphate buffer pH = 5.5 at 32 °C. **** *p* < 0.0001.

The release profile of CO from the hydrogels was comparatively investigated during 48 h (Figure 7). The release pattern was biphasic for all samples, displaying an initially faster release rate followed by a slow and progressive release for the subsequent hours. Practically, the amount of CO released after 7 h varied between 50% and 60% for the samples CS/OP_0.5_-C_1_, CS/OP_1_-C_1_, and CS/OP_2_-C_1_, depending on the porosity and the composition of the hydrogels. Then, the release was slow and continuous for the subsequent 41 h. As can be seen in Figure 7, the release profiles were not significantly different (*p* > 0.05) for the first three samples, but a clear difference (*p* < 0.00001) was observed in the case of CS/OP_1_-C_5_ (the fourth sample in Table 2). In fact, after seven hours, this hydrogel released only 8.7% from the total amount of entrapped oil, and 11.3% after the next 41 h. As expected, by increasing the initial CO concentration in the hydrogel sample, the amount of released drug decreased due to the higher hydrophobic character of the entire network. On the other hand, a high amount of incorporated oil reduced the number of empty pores (as previously shown by SEM analysis) and behaved like a barrier that prevents the penetration of dissolution media. This statement is in agreement with previously reported studies for essential oils incorporated into chitosan cryogels [66,74] or PVA/starch hydrogel membranes [75]. It must be noted that the decrease in OP content (low degree of cross-linking) enhanced the release of CO. This behavior is attributed to the larger porosity of the sample and the higher degree of swelling in the release medium (see Figure 5). A reduced degree of cross-linking means more hydrophilic groups are available for water bonding and a greater rate of diffusion of CO molecules through the hydrophilic swollen polymeric network.

Usually, the healing process of wounds can last from one month to several years and an ideal dressing must ensure the antioxidant and antibacterial effect (at least during the first 120 h after injury). With that in mind, the release profile of CO from the CS/OP_0.5_-C_1_ and CS/OP_1_-C_1_ hydrogels was followed for up to 15 days (inset of Figure 7) when the plateau pattern with a slow release of CO continued up to the end of the experiment. After 15 days, the percentages of CO released were 88.2% and 76.5% for CS/OP_0.5_-C_1_ and CS/OP_1_-C_1_, respectively. 

Considering the release pattern kinetics of CO from the four tested hydrogel samples, the slower but still high release rate in acidic conditions (useful in topical formulations [76]) showed by the CS/OP_1_-C_1_ hydrogel can be exploited for further use as dressing for wound healing. 

### 2.4. Antioxidant Activity

**Figure 8:**
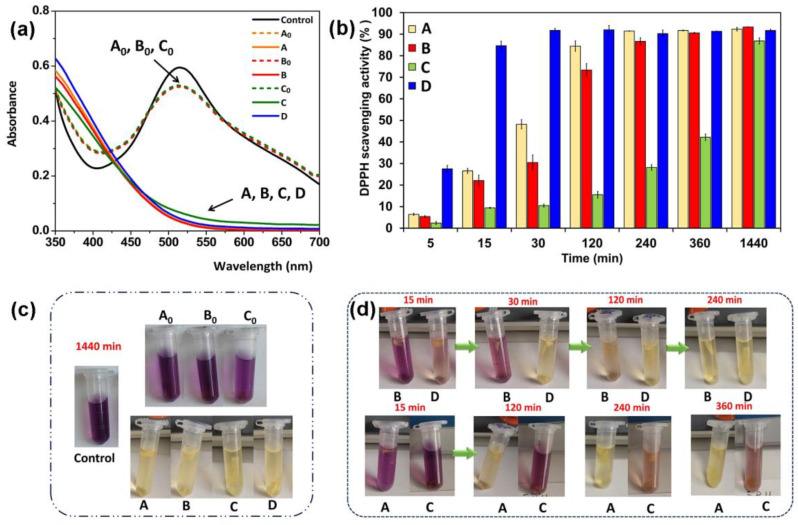
Antioxidant properties of CS/OP hydrogels: (**a**) absorbance of DPPH solution before (control) and after mixing with unloaded (A_0_, B_0_, C_0_) and CO-loaded CS/OP hydrogels (A, B, C, D); (**b**) antioxidant activity of CO-loaded CS/OP hydrogels as a function of immersion time; (**c**) photographs of the DPPH solution in the absence and presence of the hydrogels after 24 h of immersion, and (**d**) photographs of the color change of the DPPH solution over time after mixing with CO-loaded CS/OP hydrogels (A = CS/OP_0.5_-C_1_, B = CS/OP_1_-C_1_, C = CS/OP_2_-C_1_, D = CS/OP_1_-C_5_). Results are expressed as means ± standard deviation (S.D.) of three experiments (n = 3).

It is well known that CO, especially due to the eugenol component, possesses free radical scavenging activity and lipid peroxidation inhibition activity; it also induces collagen synthesis by dermal fibroblasts [49]. Moreover, this compound can inhibit reactive oxygen species, nitric oxide production, and myeloperoxidase activity in human neutrophils [77]. For this study, three samples with the best mechanical features were chosen, namely CS/OP_1_-C_1_, CS/OP_2_-C_1_, and CS/OP_1_-C_5_ (see Table 2). To assess the antioxidant activity of the hydrogels containing CO, samples with different CO content and degrees of cross-linking were tested while unloaded CS/OP hydrogels were used as controls. The DPPH scavenging assay was used to determine the antioxidant activity of free CO and CO-loaded hydrogels. The IC_50_ value of CO used in this present work was calculated as 0.01 µg/mL (Figure S4), indicating a high antioxidant potential, stronger than that found by Jirovetz et al. [78] (IC_50_ = 0.08 µg/mL). Thanks to its higher antioxidant activity, CO could be used as a potential alternative to natural antioxidants when incorporated into the polymeric matrix. It must be noted that the unloaded CS/OP hydrogels showed a slight scavenging potential towards the DPPH radical, regardless of the degree of cross-linking, calculated as less than 10% after 24 h (Figure 8a). This result is in good agreement with previously reported data from the literature [79] which attributed this property to its intrinsic activity given by hydrogen donor –OH and -NH_2_ groups. The CO-loaded hydrogels herein prepared showed radical scavenging properties in a concentration- and time-dependent mode. The scavenging activity of the tested hydrogels was in the order: CS/OP_1_-C_5_ > CS/OP_1_-C_1_ > CS/OP_2_-C_1_, and the maximum values were obtained after 24 h of incubation (82 ÷ 93%), as shown in Figure 8b. It was observed that the discoloration of DPPH intensified by increasing the CO concentration and reaction time (over 80% achieved after 5 min for CS/OP_1_-C_5_ compared with 240 min for CS/OP_1_-C_1_). In particular, the antioxidant activity significantly increased after two hours of exposure for the CS/OP_1_-C_1_ (73.4%) and CS/OP_1_-C_5_ (92.0%) samples. Also, the scavenging activity was correlated to the degree of cross-linking in the hydrogels, a DPPH reduction of 42.2% was recorded for the CS/OP_2_-C_1_ sample after 360 min compared with a 90.5% reduction ratio for CS/OP_1_-C_1_. The reduced antioxidant activity may be due to the lower CO content but it is most probably determined by the slower diffusion of CO from the denser and thicker walls of the higher cross-linked CS/OP_2_-C_1_ sample. To observe with greater certainty the free radical elimination ability of the hydrogel, Figure 8c,d show the color changes of the solutions at different times. 

Similar antioxidant properties were reported when CO-containing chitosan-based hydrogels [50] reduced the antioxidant activity by about 47%. Due to their high free radical scavenging activity, the CO-loaded hydrogels could prevent lipid peroxidation and enzyme inactivation and thus could accelerate the wound healing process.

Considering the overall physicochemical properties and the mostly in vitro release profiles, samples CS/OP_1_-C_1_, CS/OP_2_-C_1_, and CS/OP_1_-C_5_ were further characterized from the point of view of their antimicrobial performances.

### 2.5. Antibacterial Assay

**Figure 9:**
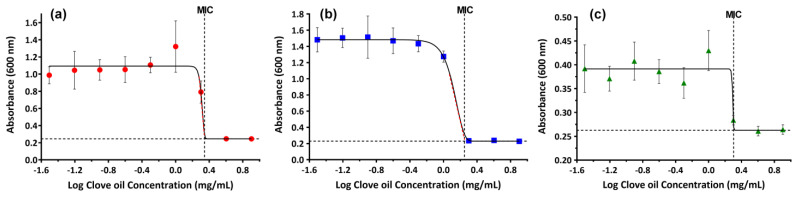
Maximum average absorbance (OD at 24 h) of *S. aureus* (**a**), *E. coli* (**b**), and *C. albicans* (**c**) at different CO concentrations. Symbols—measured OD; lines—fitted Gompertz function. Each point is the average of three separate trials, with the standard deviation as the error bars.

The inhibitory effect of CO on the growth of significant pathogens found in skin damage was studied and the minimum inhibitory concentration (MICs) values were quantified. The plots of the maximum absorbance values measured at 24 h, representing the maximum growth of bacteria, as function of the CO concentrations (Figure 9), show that, at essential oil concentrations lower than 0.5 mg/mL, bacteria growth is barely affected by the presence of CO. As the concentration of CO increased, starting from 2 mg/mL for *S. aureus* and *C. albicans*, and 1 mg/mL for *E. coli*, bacteria growth decreased until no more progression was observed. The MICs of CO against the three chosen microorganisms were obtained by fitting the average absorbance data as shown in Figure 9. The results demonstrate a similar activity against *S. aureus* and *C. albicans* (MIC of 2 mg/mL) and a slightly better activity against *E. coli* (MIC of 1.78 mg/mL). The obtained data are similar to those reported in the literature [57,80,81] and can be explained by the different composition of their cell wall structures that distinguish Gram-positive from Gram-negative bacteria. 

The disc diffusion method showed that CS/OP hydrogels without CO did not inhibit the growth of the three selected bacteria and fungi strains (Figure S5). Also, the results for the CO-loaded hydrogels were not relevant, the diffusion of CO in agar medium being slowed down by the presence of the hydrophilic–hydrophobic forces. Moreover, due to the low diffusion in solid media, the hydrogels with high antimicrobial activity (high CO content) cannot prevent the growth of bacteria. Our results are comparable to previously reported studies [82,83].

**Figure 10:**
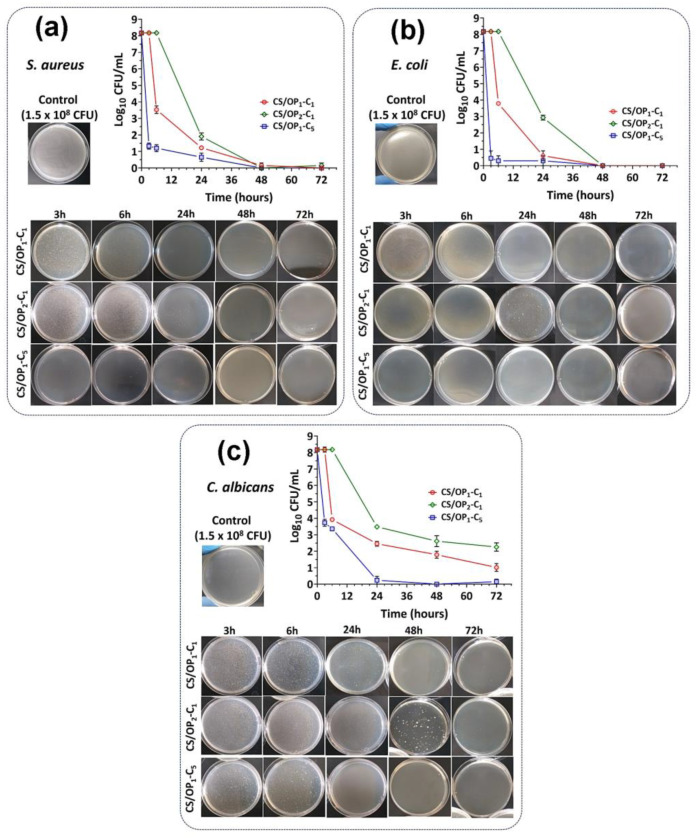
Antibacterial activity of CS/OP hydrogels containing CO after 3, 6, 24, 48, and 72 h of incubation with *S. aureus* (**a**), *E. coli* (**b**), and *C. albicans* (**c**).

Therefore, the bactericide and bacteriostatic effect of CO-loaded hydrogels was evaluated by the time-kill method, a more appropriate approach for this kind of material. The antimicrobial and antifungal effects of the hydrogels against total viable bacteria (Log CFU/mL) were measured after incubation of bacterial strains at a density of 1.5 × 10^8^ CFU/mL in the presence of hydrogels for different periods. As the results show (Figure 10), the effectiveness of the CS/OPx-Cy hydrogels was better against *E. coli* than *S. aureus*, similar to that of free CO, due to the different composition of the cell wall structure [57,80]. All hydrogels provided a significant reduction in the bacterial number indicating their good antibacterial activity. The release of the CO from the hydrogels produced a significant reduction in the viable cell count during the different incubation times. When comparing the antibacterial effect of CO-loaded hydrogels, the results showed that the antibacterial activity is affected by the CO content and the cross-linking degree of hydrogels for all bacterial strains (Figure 10). For all bacteria tested, we can conclude that the antimicrobial activity order of CO-loaded hydrogels is CS/OP_1_-C_5_ > CS/OP_1_-C_1_ > CS/OP_2_-C_1_. The CS/OP_1_-C_5_ hydrogel, containing the highest amount of CO, is the most effective in terms of the reduction in the bacterial population due to the high CO amount released. It must be noted that all three samples showed a bacteriostatic effect, since no colony formation was observed for 48 and 72 h (Figure 10a,b). The rapid effect (0.45 log_10_ reduction) of CS/OP_1_-C_5_ observed after 3 h against *E. coli* is partly due to the higher susceptibility of this strain to CO and to the superior CO content of this sample.

The antifungal activity of the hydrogels was also screened against *Candida albicans*, the fungistatic activity of CO being previously reported in the literature [84,85] at a MIC value of 3.2 mg/mL [86,87]. After 3 h of contact with the CS/OP_1_-C_5_ hydrogels, a decrease of 45% in CFU counts was evidenced, corresponding to a 99.99% reduction (Figure 10c). The CS/OP_1_-C_1_ sample began its activity after 6 h, with a 3.9 log reduction value being measured. It must be noted that only the sample with the higher CO content showed a potential fungistatic effect after 48 and 72 h (Figure 10c), for the other samples a continuous decrease in CFU counts in time was observed. Surprisingly, the hydrogel with the higher cross-linking degree and lower CO content showed a low antifungal activity; after 24 h, a growth of *C. albicans* was noticed. To conclude, the CS/OP_x_-C_y_ hydrogels presented moderate antifungal activity against *C. albicans*, the effect being influenced by the degree of CO loading, the morphology, and the degree of cross-linking, which ultimately control the diffusion of the essential oil through the polymeric matrix to bacterial encounters. 

## 3. Conclusions

The CO-loaded hydrogel emulsions were synthesized by an original procedure. First, the CO was stabilized by ultrasound emulsification in a solution of chitosan. Then, the chitosan solution embedding dispersed droplet oils was hardened by double cross-linking: covalent with macromolecular oxidized pullulan, and physical using the freeze–thaw method. The physical cross-linking was used to improve the stability of the hydrogel and to create interconnected pores after the removal of the frozen water by lyophilization. The CS/CO emulsion droplets showed excellent stability for 24 h. The hydrogels displayed a foam-like structure and the size of the CO droplets embedded in the hydrogel walls was almost identical to that found in the starting emulsions, demonstrating that the emulsion was stable enough during the preparation process. By adjusting the degree of cross-linking and the amount of CO embedded, the resulting hydrogels had excellent mechanical properties. Due to the high swelling capacity, fast shape recovery, sustained in vitro oil release performance, antioxidant and antibacterial properties, the herein prepared CO-loaded hydrogels could be promising as future wound dressing materials.

## 4. Materials and Methods

### 4.1. Materials

Low molecular weight chitosan (CS, CAS Number 9012-76-4) was provided by Aldrich GmbH (Steinheim, Germany). The degree of deacetylation of the CS was 81.26%, as determined by NMR spectroscopy [88], while the molecular weight, M_w_ = 330 kDa, was calculated from viscometric measurements according to the method described in the literature [89]. Pullulan (P, Mw = 200 Kg/mol) was purchased from Hayashibara Laboratories LTD (Okoyama, Japan), clove essential oil (CO, *Eugenia caryophyllus* leaf oil) was supplied from Bioskin-Plafaria SRL (Iasi, Romania). Sodium periodate (NaIO_4_, 99.5% (RT)), lactic acid (LA), and ethylene glycol (EG, 99.8%) were from Sigma-Aldrich Co. (St. Louis, MI, USA) and were used without further purification. 

### 4.2. Synthesis of Oxidized Pullulan 

Oxidized pullulan (OP) was prepared according to the method described by Brunnel and Shacht [39]. Pullulan (1 g, 6.17 mmol) was dissolved in 50 mL of distilled water under stirring for 24 h. An aqueous solution (5 mL) of sodium periodate was slowly added (molar ratio IO_4_^−^/anhydroglucose unit of pullulan of 1/2) and the mixture was maintained for six hours at room temperature, in the dark, under magnetic stirring. At the end of the reaction time, an equimolar quantity of ethylene glycol with respect to NaIO_4_ was added and the stirring continued for another hour. Finally, the solution was transferred to a dialysis bag (Spectra/Por membrane, MWCO = 12,000 Da) and dialyzed against distilled water for three days, changing the water every 24 h. The dialysate was recovered by freeze drying (Yield = 80%).

### 4.3. Preparation of CO-Loaded Hydrogels

#### 4.3.1. Preparation of CS/CO Emulsion

CS solutions of different concentrations (1.75, 2.0, and 2.5%, *w*/*w*) were prepared by dissolving the appropriate amounts of CS in 1% (*w*/*v*) lactic acid at room temperature under magnetic stirring overnight to obtain homogenous solutions. The pH of the CS solutions was adjusted to 5.5 using 0.1 M NaOH solution. The CS/CO emulsions were prepared by dispersing CO (1 and 5%, *w*/*w*) in 40 g of CS solution (see Table 1) under sonication using a VCX 750 ultrasonic processor (Sonics & Materials, Inc., Newtown, CT, USA) equipped with a 3 mm conical microtip extender. The sonication process was performed at 41.67 W power for two minutes in an ice bath to avoid overheating the samples. The samples were sealed and stored at room temperature (~23 °C) for characterization studies and 4 °C for 2 h before the preparation of hydrogels. 

#### 4.3.2. Preparation of CO-Loaded CS/OP Hydrogels

Stock OP solutions of different concentrations (3, 6, and 12%, *w*/*w*) were prepared by dissolving OP in distilled water and maintained at 4 °C for 2 h. Then, four hydrogel samples with the compositions shown in Table 2 were prepared by adding the appropriate amount of OP solution to the cold CS/CO emulsion (2 wt.% containing 1% or 5% (*w*/*w*) CO) so as to obtain a final CS concentration of 1.5% and a molar ratio between the NH_2_ groups of the CS and CHO groups of OP of 1/0.5, 1/1, or 1/2. The hydrogels were prepared directly in a 12 polystyrene well plate, each well containing 3 g of polymeric solution. The OP solution was slowly added, under mild stirring, and chemical cross-linking between the amino and aldehyde groups took place within two minutes. The hydrogels were kept at room temperature for 3 h and were then subjected to 6 freeze–thaw cycles (16 h at −20 °C and 8 h at 25 °C) to promote the physical cross-linking. Finally, the hydrogels were recovered by lyophilization using ALPHA 1-2 LD, Martin Christ GmbH freeze dryer, Osterode, Germany. 

The samples were coded as **CS/OP_X_-Cy** where **x** is the molar ratio between the CHO groups of cross-linker (OP) and NH_2_ groups of CS, and **y** is the CO concentration in the CS/CO emulsion used in the formulation. Reference unloaded hydrogels were prepared in similar conditions based solely on chitosan and OP (**CS/OPx**). 

### 4.4. Determination of the Degree of Oxidation

The degree of oxidation of the OP was determined according to Constantin et al. [40] by the hydroxylamine hydrochloride method [90]. In practical terms, the hydrochloride released after the reaction of 0.1 g of OP with NH_4_OH·HCl (25 mL, 0.25 M) at pH 4 was determined by potentiometric titration with a standard NaOH solution (0.1 N). The degree of oxidation (*D.O.*) expressed as moles of aldehyde groups per moles of glucosidic units of pullulan was determined by Equation (1):

(1) D.O. (mol/g)=VNaOH ×NNaOHnCHO×mM

where *V*_NaOH_ is the volume of consumed NaOH solution, *N*_NaOH_ is the concentration of NaOH solution (0.1 mol/L), *n*_CHO_ is the possible number of aldehyde groups that can be introduced onto the structural unit of pullulan (*n* = 2), *m* is the dry weight of the oxidized sample, and *M* is the molecular weight of the repeating unit of pullulan (162 g/mol).

### 4.5. Determination of Chemical Cross-Linking Degree by Ninhydrin Assay

The degree of chemical cross-linking of the CO-loaded CS/OP hydrogels was determined using the ninhydrin assay [91] by comparing the number of free amino groups of the hydrogels to the number of free amino groups on the non-cross-linked chitosan. First, in a test tube, 5 mg of lyophilized hydrogel was swollen overnight in 5 mL of 0.5% acetic acid. Then, 5 mL of freshly prepared nynhydrin reagent was added, and the mixture was heated in a water bath for 30 min at 100 °C to allow the reaction between the ninhydrin and the amino groups of CS in hydrogels [92]. After cooling the solution at room temperature, 0.5 mL was diluted with 10 mL of ethanol/water (1/1, *v*/*v*) and the absorbance at 570 nm was measured using an Evolution 201 UV-Visible Spectrometer (ThermoFisher Scientific, Walthman, MA, USA). The reference content of the free amino groups in non-cross-linked CS was determined using the same procedure for 0.1 ÷ 1 mg/mL CS solutions in 0.5% acetic acid. The solution absorbance was related to the number of free amino groups using a calibration curve obtained with glycine standard solutions [84]. Finally, the chemical cross-linking degree (*CD*%) was calculated according to Equation (2):

(2) $$CD\;\left(\%\right)=\frac{NH_{2\left(\mathrm{CS}\right)}-NH_{2\left(\mathrm{Hydrogel}\right)}}{NH_{2\left(\mathrm{CS}\right)}}\times 100$$

where *NH*_2(CS)_ is the number of free amino groups of non-cross-linked chitosan and *NH*_2(hydrogel)_ is the number of free amino groups measured for cross-linked hydrogel samples.

### 4.6. Characterization

#### 4.6.1. CS/CO Emulsion 

The particle size distribution and mean droplet diameter (nm) of the CS/CO emulsions were measured by dynamic light scattering (DLS) using a Zetasizer Nano-ZS diffractometer (Malvern Instruments Ltd., Worcestershire, UK). Before the measurements were conducted, the samples were diluted with distilled water to an appropriate concentration (1:10) to avoid multiple scattering effects.

The physical stability of the emulsion was evaluated by DLS measurements at regular time intervals and by visual observation based on the resistance of the emulsion system to coalescence for four days. The freshly made emulsion was poured into a 10 mL graduated cylinder with a stopper and stored at room temperature in the dark. The height of the opaque emulsion phase was measured daily for four days (*h*_t_) and compared to the initial emulsion height (*h*_0_) to determine sedimentation stability (*S*) using Equation (3):

(3) $$S\;\left(\%\right)=\frac{h_{0}-h_{t}}{h_{0}}\times 100$$

#### 4.6.2. CO-Loaded CS/OP Hydrogels

*Structural characterization*

The infrared spectra of the CS/OP hydrogels with and without CO were recorded using a Bruker Vertex 70 (Billerica, MA, USA) spectrometer equipped with attenuated total reflection (ATR) with ZnSe crystal, in the wave number range from 4000 to 600 cm^−1^ at room temperature with a resolution of 2 cm^−1^.

*Morphology and porosity*

The cross-section morphology of the CO-loaded CS/OP hydrogels was investigated using a Verios G4 UC Scanning Electron Microscope (Thermo Scientific, Brno, Czech Republic). The samples were coated with 10 nm platinum, at 30 mA, using a Leica EM ACE200 Sputter coater to ensure the proper electrical conductivity and to prevent charge build-up during exposure to the electron beam. 

The porosity of the hydrogels was estimated using a slightly modified solvent immersion method [93]. Freeze-dried hydrogels of the same shape were weighed, and then immersed in ethanol (99.5%). After 5 min of immersion, the samples were taken out and the excess alcohol was removed. The samples were weighed and the porosity was calculated following Equation (4):

(4) $$Porosity\;\left(\%\right)=\frac{\left(W_{2}-W_{1}\right)}{\rho V}\times 100$$

where *W*_2_ and *W*_1_ represent the weight of the hydrogels before and after immersion in alcohol, *V* is the volume of the hydrogels before immersion, and ρ is the density of ethanol. All experiments were performed in triplicate.

*Gel fraction*

To evaluate the percentage of polymers (CS and OP) involved in the physical cross-linking reaction, the gel fraction (GF, %) was determined. In other words, GF can be assimilated with a degree of cross-linking. In this respect, the CO-loaded CS/OP hydrogels were freeze-dried and weighed (*W*_i_, g). Then, the hydrogels were purified with distilled water to remove the non-cross-linked polymer chains. The resulting hydrogels were freeze-dried again, and weighed (*W*_p_, g). The gel fraction (*GF*, %) was calculated using Equation (5):

(5) $$GF\left(\%\right)=\frac{W_{p}}{W_{i}}\times 100$$

where *W*_p_ is the weight of the washed sample in the dried condition; *W*_i_ is the weight of the dried sample before the washing step.

*The loading efficiency*

The total CO content of the CS/OP hydrogels was determined using the alcoholic extract method. Briefly, 50 mg of the crushed sample was immersed in 4 mL of an ethanol/water mixture (70/30, *v*/*v*) and kept for 24 h in the dark under gentle shaking and at room temperature. Then, the absorbance was measured at 280 nm using a UV–vis spectrophotometer (Thermo Fisher Scientific Evolution 201, Waltham, MA, USA). The total CO content (*LC*) was calculated from the CO calibration curve, determined using the ethanol/water CO solutions with various concentrations (5 ÷ 100 μg/mL, y = 0.0158x; R^2^ = 0.9996), and the results were expressed as mg CO/g hydrogel. The loading efficiency (*LE*) was calculated using the following Equation (6):

(6) $$LE\;\left(\%\right)=\frac{CO_{a}}{CO_{i}}\times 100$$

where *CO*_a_ represents the actual CO content determined by UV–vis spectrometry and *CO*_i_ is the theoretical amount of CO used for the formulation preparation.

### 4.7. Swelling Behavior

The swelling of the hydrogels was studied in phosphate-buffered solution (PBS) with a pH = 5.5 and kept at 32 °C, to simulate the skin environment. The dried hydrogels were weighed (*W*_d_, g) and immersed in the PBS solution. After different time intervals the hydrogels were withdrawn and, after careful removal of the superficial solution with wet filter paper, the samples were weighed again (*W*_t_, g). The swelling ratio (*SR*) was calculated following Equation (7):

(7) $$SR\left(\%\right)=\frac{\left(W_{t}-W_{d}\right)}{W_{d}}\times 100$$

After 24 h, when the equilibrium was reached, the samples were weighed (*W*_e_, g), and the equilibrium swelling ratio (*SR*_eq_) was calculated according to Equation (8):

(8) $$SR_{eq}\left(\%\right)=\frac{\left(W_{e}-W_{d}\right)}{W_{d}}\times 100$$

### 4.8. Mechanical Properties

The mechanical properties were investigated at room temperature using Texture Analyser (Brookfield Texture PRO CT3^®^, Brookfield Engineering Laboratories Inc., Middleborough, MA, USA), following the ASTM D882 standard. The compression tests were performed following the protocol described in reference [94] with slight modifications. Circular CO-loaded CS/OP hydrogel samples (20 mm diameter and 8 mm height) were previously saturated in PBS at pH 5.5 for 30 min, and then compressed until 70% deformation using a trigger load of 0.067 N at a speed rate of 0.2 mm/s. The elastic modulus (E, kPa) was calculated from the slope between 2 and 10% of the linear part of the stress–strain curves. 

Furthermore, successive loading–unloading cyclic tests with compression strain were carried out to study the reversible behavior of the hydrogels. A 20 s relaxation time was fixed between the four loading–unloading cycles. 

### 4.9. In Vitro Clove Oil Release Study

The in vitro release of CO from the hydrogels was carried out in PBS solution (pH 5.5) at 32 °C in order to simulate physiological dermal conditions by immersing 50 mg of the CO-loaded hydrogels in 4 mL of the release medium without stirring. At predetermined time intervals, 3 mL of the solution was withdrawn and the same volume of fresh buffer was added [57,82]. The amount of CO released was calculated from the UV absorbance peak at 280 nm using the same UV–vis spectrophotometer. The standard calibration curve of CO was obtained by measuring the absorbance of the CO solutions in the concentration range (5–100 μg/mL) prepared from a stock solution in ethanol (1 mg/mL) and subsequently dilutions in PBS (pH 5.5). Each experiment was conducted in triplicate. The calibration curve of CO was then plotted with absorbance on the y-axis and the CO concentration on the x-axis (y = 0.0186x, R^2^ = 0.9984).

### 4.10. Antioxidant Activity

The antioxidant activity was spectrophotometrically evaluated through the free radical scavenging capacity using a 2,2-diphenyl-l-picrylhydrazil (DPPH) assay. The methodology described in the literature [57,95] was used with slight modifications in order to assess the DPPH free radical scavenging capacity of the free CO, CO-loaded and unloaded CS/OP hydrogel samples. In short, 1 mL of ethanolic solution of CO with different concentrations (1.25 × 10^−3^ ÷ 0.634 μg CO/mL) was mixed with 0.5 mL of ethanolic solution of DPPH (0.1 mM). The solutions were shaken and incubated for 30 min in darkness at room temperature. The DPPH scavenging effect was calculated using Equation (9) considering the absorbance value taken at a wavelength of 515 nm by means of a UV–vis spectrophotometer:

(9) $$\mathrm{DPPH}˙\;\left(\%\right)=\left(1-\frac{A_{s}}{A_{c}}\right)\times 100$$

where *A*_c_ is the absorbance of the control sample (a mixture of 1 mL of ethanol with 0.5 mL DPPH˙ solution) and *A*_s_ is the absorbance of the tested sample. The low absorbance of the reaction mixture indicates a high DPPH˙ free radical scavenging activity. IC_50_, which denotes the concentration (μg/mL) of CO required to reduce the initial concentration of DPPH radicals by 50%, was also calculated.

The free radical scavenging activity of the hydrogels was determined by immersing ~5 mg of each sample into 1.5 mL of DPPH ethanolic solution (0.1 mM). The solutions were shaken and incubated for different times (5, 15, and 30 min and 2, 4, 6, and 24 h) in darkness and at room temperature. The DPPH scavenging activity was determined using the method described above for free CO.

### 4.11. Antimicrobial Activity

Three standardized bacterial cultures of *Staphylococcus aureus* ATCC 25923 (*S. aureus*), *Escherichia coli* ATCC 25922 (*E. coli*), and *Candida albicans* ATCC 90028 (*C. albicans*) were used to assess the antimicrobial and antifungal activity of CO and the unloaded and loaded CS/OP hydrogels.

#### 4.11.1. MIC Determination of the CO

The minimal inhibitory concentrations (MICs) were measured by the broth microdilution method [96]. The inocula were prepared at a final density adjusted to the 0.5 McFarland turbidity standard (10^8^ colony-forming units CFU/mL). The essential clove oil was individually dissolved in ethanol solution (99.5%, *w*/*v*) at final concentrations in the range of 8 ÷ 3.125 × 10^−1^ mg/mL. Each essential oil solution (20 µL) was dispensed into 30 μL of bacterial suspension and then inoculated with 150 µL of the broth medium (Mueller Hinton Broth, Oxford, UK), mixed with a micro-pipettor and incubated at 37 °C for 24 h. The positive control consisted of 10 μL of bacterial culture inoculated in 190 μL of Mueller–Hinton Broth. The sterile broth medium (190 µL) represented the negative control. Bacterial growth was spectrophotometrically measured at λ = 630 nm using a Stat Fax 3200 microplate reader (Awareness Technology, Palm City, FL, USA). Two measurements were performed: the first before incubation and the second after incubation. The lowest concentration of CO that inhibited the visible growth of microorganisms (MIC) was determined by fitting the maximum average absorbance values with the modified Gompertz equation [97] using GraphPad Prism 8.02 (263) software (San Diego, CA, USA). 

#### 4.11.2. Difusimetric Test of Unloaded and CO-Loaded CS/OP Hydrogels

The antimicrobial and antifungal activity of the unloaded and CO-loaded CS/OP hydrogels was evaluated by the Kirby–Bauer test [98]. For this, standardized suspensions of bacterial cultures of *S. aureus*, *E. coli*, and *C. albicans* were prepared to a cell density corresponding to the 0.5 McFarland turbidity standard (1.5 × 10^8^ CFU/mL). From every microbial suspension, one mL was pulled out and dispersed into Petri dishes (90 mm) over which a Mueller–Hinton agar (Oxoid Ltd., Basingstoke, UK) for *S. aureus* and *E. coli,* and a potato–dextrose agar (Oxoid Ltd., Basingstoke, UK) for *C. albicans* was spread. 

Hydrogels cut into square shapes (7 × 7 mm^2^ and 4 mm height, ~7 mg each) were hydrated with 50 µL of sterile distilled water and then placed on the agar plate containing bacterial suspension and incubated at 37 °C for 24 h. The antimicrobial activity was evaluated by measuring the diameter of the inhibition zones formed around each sample. All experiments were performed in triplicate.

#### 4.11.3. Time-Kill Studies

For the time-kill method [99], the hydrogel samples were distributed in sterile vials, over which 5 mL of bacterial suspension (1.5 × 10^8^ CFU/mL) was added. The samples were incubated at 37 °C for different times (3, 6, 24, 48, and 72 h). After each incubation time, the vials were homogenized by vortexing for 30 s at 1000 rpm, and then 1 mL of bacterial suspension was taken out and spread in sterile Petri dishes; melted culture medium was added on top of each and cooled to 45 °C. After toughening the culture medium, the samples were incubated for 24 h at 37 °C to allow the growth of the remaining viable microbial cells. After this time, all the microbial colonies formed in the culture medium (CFU = colony forming units) were counted, transformed into logarithmic values (Log10), and time-kill curves were constructed by plotting log10 CFU/mL against time (h). Each assay included a positive growth control (C+) represented by 1 mL of bacterial suspension with a turbidity of 0.5 McFarland standard (1.5 × 10^8^ CFU/mL). Experiments were carried out in triplicate. 

## Figures and Tables

**Table 1:** The main properties of CS/CO emulsions.

Sample Code	CS Conc. (%, *w*/*w*)	CO Conc. (%, *w*/*w*)	Size (nm)/Storage Time (Hours)					
			0 h	1 h	2 h	3 h	4 h	24 h
CS/CO 1	1.75	1	906 ± 13	1163 ± 27	1277 ± 27	1430 ± 26	1408 ± 31	1636 ± 20
CS/CO 2	2		1149 ± 24	1196 ± 47	1410 ± 26	1436 ± 14	1565 ± 35	1822 ± 31
CS/CO 3	2.5		1165 ± 17	1305 ± 16	1473 ± 28	1589 ± 19	1920 ± 36	2235 ± 25
CS/CO 4	2	5	2205 ± 25	2372 ± 16	2455 ± 26	2690 ± 18	2981 ± 72	3157 ± 35

**Table 2:** The composition and main characteristics of the samples investigated in the present study.

Sample Code	Initial Mixture			Final Composition			
	CS/CO Emulsion Type	Molar Ratio NH_2_/CHO	W_CS_/W_OP_/W_CO_ (%, *w*/*w*)	GF (%)	C.D. (%)	LC (%, *w*/*w*)	Efficiency (%)
CS/OP_0.5_-C_1_	CS/CO 2	1/0.5	61.7/19.2/19.1	67.6 ± 1.4	5.3 ± 0.8	12.8 ± 0.7	66.9 ± 6.0
CS/OP_1_-C_1_		1/1	51.8/32.1/16.1	75.5 ± 1.8	11.4 ± 1.6	14.1 ± 1.2	87.8 ± 7.5
CS/OP_2_-C_1_		1/2	39.2/48.6/12.2	63.9 ± 1.1	35.9 ± 2.5	8.7 ± 0.4	71.7 ± 2.1
CS/OP_1_-C_5_	CS/CO 4	1/1	31.5/19.6/48.9	53.1 ± 2.4	7.6 ± 0.5	46.5 ± 1.7	95.2 ± 3.8

W_CS_, W_OP_, and W_CO_ represent the weight percentages of CS (as lactate), OP, and CO, respectively, in the polymeric mixture; C.D. is the chemical cross-linking degree determined by ninhydrin assay and calculated using Equation (2) and LC is the loading CO content determined by UV–vis spectrometry. Measurements were performed in triplicate (mean ± standard deviation).

## Data Availability

The data presented in this study are available on request from the corresponding author.

## Supplementary Materials

The following supporting information can be downloaded at: https://www.mdpi.com/article/10.3390/gels10040227/s1, Figure S1: FT-IR spectrum of clove oil; Figure S2: FT-IR spectrum of chitosan lactate; Figure S3: FT-IR spectrum of oxidized pullulan; Figure S4: DPPH radical scavenging capacity of clove oil; Figure S5: Antibacterial activity of un-loaded and CO-loaded CS/OP hydrogels against *S. aureus*, *E. coli*, and *C. albicans* according to well diffusion assay.

## References

[1] Tariq S., Wani S., Rasool W., Shafi K., Bhat M.A., Prabhakar A., Shalla A.H., Rather M.A. (2019). A comprehensive review of the antibacterial, antifungal and antiviral potential of essential oils and their chemical constituents against drug-resistant microbial pathogens. Microb. Pathog..

[2] Mazutti da Silva S.M., Rezende Costa C.R., Martins Gelfuso G., Silva Guerra E.N., De Medeiros Nóbrega Y.K., Gomes S.M., Pic-Taylor A., Fonseca-Bazzo Y.M., Silveira D., Magalhães P.O. (2019). Wound Healing Effect of Essential Oil Extracted from *Eugenia dysenterica* DC (Myrtaceae) Leaves. Molecules.

[3] Tit D.M., Bungau S.G. (2023). Antioxidant Activity of Essential Oils. Antioxidants.

[4] Yammine J., Chihib N.E., Gharsallaoui A., Ismail A., Karam L. (2023). Advances in essential oils encapsulation: Development, characterization and release mechanisms. Polym. Bull..

[5] Chen L.-H., Cheng L.-C., Doyle P.S. (2020). Nanoemulsion-Loaded Capsules for Controlled Delivery of Lipophilic Active Ingredients. Adv. Sci..

[6] Purwanti N., Zehn A.S., Pusfitasari E.D., Khalid N., Febrianto E.Y., Mardjan S.S., Kobayashi A.I. (2018). Emulsion stability of clove oil in chitosan and sodium alginate matrix. Int. J. Food Prop..

[7] Wang X.-Y., Heuzey M.-C. (2016). Chitosan-based conventional and pickering emulsions with long-term stability. Langmuir.

[8] Niu F., Pan W., Su Y., Yang Y. (2016). Physical and Antimicrobial Properties of Thyme Oil Emulsions Stabilized by Ovalbumin and Gum Arabic. Food Chem..

[9] Ma Q., Davidson P.M., Zhong Q. (2016). Antimicrobial properties of microemulsions formulated with essential oils, soybean oil, and Tween 80. Int. J. Food Microbiol..

[10] Ghosh V., Saranya S., Mukherjee A., Chandrasekaran N. (2013). Antibacterial microemulsion prevents sepsis and triggers healing of wound in wistar rats. Colloids Surf. B.

[11] Yang Q.-Q., Sui Z., Lu W., Corke H. (2021). Soybean lecithin-stabilized oil-in-water (o/w) emulsions increase the stability and in vitro bioaccessibility of bioactive nutrients. Food Chem..

[12] Campolo O., Giunti G., Laigle M., Michel T., Palmeri V. (2020). Essential oil-based nano-emulsions: Effect of different surfactants, sonication and plant species on physicochemical characteristics. Ind. Crops Prod..

[13] Chiriac A.P., Rusu A.G., Nita L.E., Chiriac V.M., Neamtu I., Sandu A. (2021). Polymeric carriers designed for encapsulation of essential oils with biological activity. Pharmaceutics.

[14] Huerta R.R., Silva E.K., El-Bialy T., Saldana M.D.A. (2020). Clove essential oil emulsion-filled cellulose nanofiber hydrogel by high-intensity ultrasound technology for tissue engineering applications. Ultrason. Sonochem..

[15] Wang J., Li Y., Gao Y., Xie Z., Zhou M., He Y., Wu H., Zhou W., Dong X., Yang Z. (2018). Cinnamon oil-loaded composite emulsion hydrogels with antibacterial activity prepared using concentrated emulsion templates. Ind. Crops Prod..

[16] Wang F., Sun Q., Li Y., Xu R., Li R., Wu D., Huang R., Yang Z., Li Y. (2024). Hydrogel encapsulating wormwood essential oil with broad-spectrum antibacterial and immunomodulatory properties for infected diabetic wound healing. Adv. Sci..

[17] Lu G., Shen X., Xiao D., Rong L., Mao Z., Wang B., Sui X., Zhao M., Feng X. (2022). Antibacterial thyme oil-loaded zwitterionic emulsion hydrogels. J. Mater. Chem. B.

[18] Hu T., Xu Y., Xu G., Pan S. (2022). Sequence-selected C_13_-Dipeptide self-assembled hydrogels for encapsulation of Lemon Essential Oil with antibacterial activity. J. Agric. Food Chem..

[19] Cai K., Liu Y., Yue Y., Liu Y., Guo F. (2023). Essential oil nanoemulsion hydrogel with anti-biofilm activity for the treatment of infected wounds. Polymers.

[20] Catanzano O., Straccia M.C., Miro A., Ungaro F., Romano I., Mazzarella G., Santagata G., Quaglia F., Laurienzo P., Malinconico M. (2015). Spray-by-spray in situ cross-linking alginate hydrogels delivering a tea tree oil microemulsion. Eur. J. Pharm. Sci..

[21] Li X., Gao Y., Li Y., Li Y., Liu H., Yang Z., Wu H., Hu Y. (2022). Formation of cinnamon essential oil/xanthan gum/chitosan composite microcapsules basing on Pickering emulsions. Colloid Polym. Sci..

[22] Low W.L., Kenward M.A.K., Amin M.C.I.M., Martin C. (2016). Ionically Crosslinked Chitosan Hydrogels for the Controlled Release of Antimicrobial Essential Oils and Metal Ions for Wound Management Applications. Medicines.

[23] Rusu A.G., Nita L.E., Rosca I., Croitoru A., Ghilan A., Tartau-Mititelu L., Grigoras A.V., Cretu B.-E.-B., Chiriac A.P. (2023). Alginate-based hydrogels enriched with lavender essential oil: Evaluation of physicochemical properties, antimicrobial activity, and in vivo biocompatibility. Pharmaceutics.

[24] Bhattarai N., Gunn J., Zhang M. (2010). Chitosan-based hydrogels for controlled, localized drug delivery. Adv. Drug Deliv. Rev..

[25] Hoffman A.S. (2002). Hydrogels for biomedical applications. Adv. Drug Deliv. Rev..

[26] Maurya A., Singh V.K., Das S., Prasad J., Kedia A., Upadhyay N., Dubey N.K., Dwivedy A.K. (2021). Essential Oil Nanoemulsion as Eco-Friendly and Safe Preservative: Bioefficacy against Microbial Food Deterioration and Toxin Secretion, Mode of Action, and Future Opportunities. Front. Microbiol..

[27] dos Santos M.K., Kreutz T., Danielli L.J., De Marchi J.G.B., Pippi B., Koester L.S., Fuentefria A.M., Limberger R.P. (2020). A chitosanhydrogel-thickened nanoemulsion containing *Pelargonium graveolens* essential oil for treatment of vaginal candidiasis. J. Drug Deliv. Sci. Technol..

[28] Barradas T.N., Senna J.P., Cardoso S.A., Nicoli S., Padula C., Santi P., Rossi F., de Holanda e Silva K.G., Mansur C.R.E. (2017). Hydrogel-thickened nanoemulsions based on essential oils for topical delivery of psoralen: Permeation and stability studies. Eur. J. Pharm. Biopharm..

[29] Nasr A.M., Aboelenin S.M., Alfaifi M.Y., Shati A.A., Elbehairi S.E., Elshaarawy R.F.M., Abd Elwahab N.H. (2022). Quaternized chitosan thiol hydrogel-thickened nanoemulsion: A multifunctional platform for upgrading the topical applications of virgin olive oil. Pharmaceutics.

[30] Saha R., Tayalia P. (2022). Clove oil-incorporated antibacterial gelatin–chitosan cryogels for tissue engineering: An in vitro study. ACS Biomater. Sci. Eng..

[31] Mirzaei B.A., Ramazani A.S.A., Shafiee M., Danaei M. (2013). Studies on Glutaraldehyde Crosslinked Chitosan Hydrogel Properties for Drug Delivery Systems. Int. J. Polym. Mater..

[32] Zandraa O., Ngwabebhoh F.A., Patwa R., Nguyen H.T., Motiei M., Saha N., Saha T., Saha P. (2021). Development of dual crosslinked mumio-based hydrogel dressing for wound healing application: Physico-chemistry and antimicrobial activity. Int. J. Pharm..

[33] Hu K., Jia E., Zhang Q., Zheng W., Sun R., Qian M., Tan Y., Hu W. (2023). Injectable carboxymethyl chitosan-genipin hydrogels encapsulating tea tree oil for wound healing. Carbohydr. Polym..

[34] Ngwabebhoh F.A., Zandraa O., Patwa R., Saha N., Capakova Z., Saha P. (2021). Self-crosslinked chitosan/dialdehyde xanthan gum blended hypromellose hydrogel for the controlled delivery of ampicillin, minocycline and rifampicin. Int. J. Biol. Macrom..

[35] George D., Maheswari P.U., Begum K.M.M.S., Arthanareeswaran G. (2019). Biomass-derived dialdehyde cellulose cross-linked chitosan-based nanocomposite hydrogel with phytosynthesized Zinc oxide nanoparticles for enhanced curcumin delivery and bioactivity. J. Agric. Food Chem..

[36] Singh R.S., Kaur N., Kennedy J.F. (2015). Pullulan and pullulan derivatives as promising biomolecules for drug and gene targeting. Carbohydr. Polym..

[37] Coltelli M.-B., Danti S., De Clerck K., Lazzeri A., Morganti P. (2020). Pullulan for Advanced Sustainable Body- and Skin-Contact Applications. J. Funct. Biomater..

[38] De Nooy A.E.J., Besemer A.C., Van Bekkum H., Van Dijk J.A.P.P., Smit J.A.M. (1996). TEMPO-mediated oxidation of pullulan and influence of ionic strength and linear charge density on the dimensions of the obtained polyelectrolyte chains. Macromolecules.

[39] Brunnel D., Shacht E. (1993). Chemical modification of pullulan. 1. Periodate oxidation. Polymer.

[40] Constantin M., Spiridon M., Ichim D.L., Daraba O.M., Suflet D.M., Ignat M., Fundueanu G. (2023). Synthesis, biological and catalytic activity of silver nanoparticles generated and covered by oxidized pullulan. Mat. Chem. Phys..

[41] Baron R.I., Duceac I.A., Morariu S., Bostanaru-Iliescu A.-C., Coseri S. (2022). Hemostatic cryogels based on oxidized pullulan/dopamine with potential use as wound dressings. Gels.

[42] Li X., Xue W., Liu Y., Li W., Fan D., Zhu C., Wang Y. (2016). HLC/pullulan and pullulan hydrogels: Their microstructure, engineering process and biocompatibility. Mater. Sci. Eng. C.

[43] Wang Y., Guo Z., Qian Y., Zhang Z., Lyu L., Wang Y., Ye F. (2019). Study on the electrospinning of gelatin/pullulan composite nanofibers. Polymers.

[44] Pandey V.K., Srivastava S., Ashish, Dash K.K., Singh R., Dar A.H., Singh T., Farooqui A., Shaikh A.M., Kovacs B. (2024). Bioactive properties of clove (*Syzygium aromaticum*) essential oil nanoemulsion: A comprehensive review. Helyon.

[45] Hameed M., Rasul A., Waqas M.K., Saadullah M., Aslam N., Abbas G., Latif S., Afzal H., Inam S., Akhtar Shah P. (2021). Formulation and Evaluation of a Clove Oil-Encapsulated Nanofiber Formulation for Effective Wound-Healing. Molecules.

[46] Rojas J., Cabrera S., Benavides J., Lopera Y., Yarce C.J. (2021). Lipidic matrixes containing clove essential oil: Biological activity, microstructural and textural studies. Molecules.

[47] Barradas T.N., de Holanda e Silva K.G. (2021). Nanoemusions of essential oils to improve solubility, stability and permeability: A review. Environ. Chem. Lett..

[48] Alam P., Ansari M.J., Anwer M.K., Raish M., Kamal Y.K., Shakeel F. (2017). Wound healing effects of nanoemulsion containing clove essential oil. Artif. Cellsnanomed. Biotechnol..

[49] Khunkitti W., Veerapan P., Hahnvajanawong C. (2012). In vitro bioactivities of clove buds oil (*Eugenia caryophyllata*) and its effect on dermal fibroblast. Int. J. Pharm. Pharm. Sci..

[50] Stoleru E., Dumitriu R.P., Ailiesei G.L., Yilmaz C., Brebu M. (2022). Synthesis of bioactive materials by in situ one-step direct loading of *Syzygium aromaticum* essential oil into chitosan-based hydrogels. Gels.

[51] Sapei L., Naqvi M.A., Rousseau D. (2012). Stability and release properties of double emulsions for food applications. Food Hydrocoll..

[52] Purwanti N., Ichikawa S., Neves M.A., Uemura K., Nakajima M., Kobayashi I. (2016). β-lactoglobulin as Food Grade Surfactant for Clove Oil-in-Water and Limonene-in-Water Emulsion Droplets Produced by Microchannel Emulsification. Food Hydrocoll..

[53] Hosseini S.F., Rezaei M., Zandi M., Farahmandghavi F. (2015). Bio-based composite edible films containing *Origanum vulgare* L. essential oil. Ind. Crops Prod..

[54] Yang L., Paulson A.T. (2000). Effects of lipids on mechanical and moisture barrier properties of edible gellan film. Food Res. Int..

[55] Pelin I.M., Popescu I., Calin M., Rebleanu D., Voicu G., Ionita D., Zaharia M.M., Constantin M., Fundueanu G. (2023). Tri-component hydrogel as template for nanocrystalline hydoxyapatite deposition using alternate soaking method for bone tissue engineering applications. Gels.

[56] Duceac I.A., Coseri S. (2022). Chitosan Schiff-base hydrogels-A critical perspective review. Gels.

[57] Suflet D.M., Popescu I., Pelin I.M., David G., Serbezeanu D., Rimbu C.M., Daraba O.M., Enache A.A., Bercea M. (2022). Phosphorylated curdlan gel/polyvinyl alcohol electrospun nanofibres loaded with clove oil with antibacterial activity. Gels.

[58] Tarhan I., Bakir M.R., Kalkan O., Yontem M., Kara H. (2022). Rapid determination of adulteration of clove essential oil with benzyl alcohol and ethyl acetate: Towards quality control analysis by FTIR with chemometrics. Vib. Spectrosc..

[59] Wang L.-H., Sung W.-C. (2011). Rapid evaluation and quantitative analysis of eugenol derivatives in essential oils and cosmetic formulations on human skin using attenuated total reflectance–infrared spectroscopy. J. Spectrosc..

[60] Taraj K., Andoni A., Ylli F., Ylli A., Hoxha R., Llupa J., Malollari I. (2019). Spectroscopic investigation of *Syzygium aromaticum* L. oil by water distillation extraction. J. Int. Environ. Appl. Sci..

[61] Qiao C., Ma X., Wang X., Liu L. (2021). Structure and properties of chitosan films: Effects of the type of solvent acid. LWT.

[62] Pavoni J.M.F., Luchese C.L., Tessaro I.C. (2019). Impact of acid type for chitosan dissolution on the characteristics and biodegradability of cornstarch/chitosan based films. Macromolecules.

[63] Păucean A., Vodnar D.C., Mureșan V., Fetea F., Ranga F., Man S.M., Muste S., Socaciu C. (2017). Monitoring lactic acid concentrations by infrared spectroscopy: A new developed method for *Lactobacillus* fermenting media with potential food applications. Acta Aliment..

[64] Hoffmann B., Seitz D., Mencke A., Kokott A., Ziegler G. (2009). Glutaraldehyde and oxidized dextran as crosslinker reagents for chitosan-based scaffolds for cartilage tissue engineering. J. Mat. Sci. Mat. Med..

[65] Niamsa N., Baimark Y. (2009). Preparation and characterization of highly flexible chitosan films for use as food packaging. Am. J. Food Technol..

[66] Bölgen N., Demir D., Yalçin M.S., Özdemir S. (2020). Development of *Hypericum perforatum* oil incorporated antimicrobial and antioxidant chitosan cryogel as a wound dressing material. Int. J. Biol. Macromol..

[67] Gottrup F. (2017). Oxygen therapies for wound healing: EWMA findings and recommendations. Wounds Int..

[68] Avalle M., Belingardi G., Montanini R. (2001). Characterization of polymeric structural foams under compressive impact loading by means of energy-absorption diagram. Int. J. Impact Eng..

[69] Di Muzio L., Sergi C., Carriero V.C., Tirillo J., Adrover A., Messina E., Gaetani R., Petralito S., Casadei M.A., Paolicelli P. (2023). Gelatin-based spongy and compressive resistant cryogels with shape recovery ability as ideal scaffolds to support cell adhesion for tissue regeneration. React. Funct. Polym..

[70] Yetiskin B., Okay O. (2022). Silk fibroin cryogel building adaptive organohydrogels with switching mechanics and viscoelasticity. ACS Appl. Polym. Mater..

[71] Ma X.M., Li R., Ren J., Lv X.C., Zhao X.H., Ji Q., Xi Y.Z. (2017). Restorable, high-strength poly(Nisopropylacrylamide) hydrogels constructed through chitosan-based dual macro-cross-linkers with rapid response to temperature jumps. RSC Adv..

[72] Du H., Ji Q., Xing Y., Ma X., Xia Y. (2023). A general route to strong, conductive and antibacterial curdlan-based purely natural eutectohydrogels with self-assembled layer-by-layer network structure. Carbohydr. Polym..

[73] Tanpichai S., Oksman K. (2016). Cross-linked nanocomposite hydrogels based on cellulose nanocrystals and PVA: Mechanical properties and creep recovery. Compos. Part A.

[74] Dinu M.V., Gradinaru A.C., Lazar M.M., Dinu I.A., Raschip I.E., Ciocarlean N. (2021). Physically cross-linked chitosan cryogels entrapping *Thymus vulgaris* essential oil with enhanced mechanical, antioxidant and antifungal properties. Macromolecules.

[75] Altaf F., Niazi M.B.K., Jahan Z., Ahmad T., Akram M.A., Safdar A., Butt M.S., Noor T., Sher F. (2021). Synthesis and characterization of PVA/starch hydrogel membranes incorporating essential oils aimed to be used in wound dressing applications. J. Polym. Environ..

[76] Ghorbani F.M., Kaffashi B., Shokrollahi P., Seyedjafari E., Ardeshirylajimi A. (2015). PCL/chitosan/Zn-doped nHA electrospun nanocomposite scaffold promotes adipose derived stem cells adhesion and proliferation. Carbohydr. Polym..

[77] Pérez-Rosés R., Risco E., Vila R., Peñalver P., Cañigueral S. (2016). Biological and Nonbiological Antioxidant Activity of Some Essential Oils. J. Agric. Food Chem..

[78] Jirovetz L., Buchbauer G., Stoilova I., Stoyanova A., Krastanov A., Schmidt E. (2006). Chemical composition and antioxidant properties of clove leaf essential oil. J. Agric. Food Chem..

[79] Xia W., Liu P., Zhang J., Chen J. (2011). Biological activities of chitosan and chitooligosaccharides. Food Hydrocoll..

[80] Akturk A. (2023). Enrichment of Cellulose Acetate Nanofibrous Scaffolds with Retinyl Palmitate and Clove Essential Oil for Wound Healing Applications. ACS Omega.

[81] Nzeako B., Al-Bushra L. (2008). Comparative studies of antimycotic potential of thyme and clove oil extracts with antifungal antibiotics on *Candida albicans*. Afr. J. Biotechnol..

[82] Pelin I.M., Silion M., Popescu I., Rimbu C.M., Fundueanu G., Constantin M. (2023). Pullulan/poly(vinyl alcohol) hydrogels loaded with Calendula officinalis extract: Design and in vitro evaluation for wound healing applications. Pharmaceutics.

[83] Mostaghimi M., Majdinasab M., Hosseini S.M.H. (2022). Characterization of Alginate hydrogel beads loaded with Thyme oil and Clove Essential oil nanoemulsions. J. Polym. Environ..

[84] Chee H.Y., Lee M.H. (2007). Antifungal activity of clove essential oil and its volatile vapour against dermatophytic fungi. Microbiology.

[85] Ali B.A., Ibrahim O.M.S. (2023). Antifungal activity of clove (*Syzygium aromaticum*) essential oil extract against induced topical skin infection by *Candida albicans* in mice in vivo. Egypt. J. Hosp. Med..

[86] Gucwa K., Milewski S., Dymerski T., Szweda P. (2018). Investigation of the antifungal activity and mode of action of *Thymus vularis*, *Citrus limonum*, *Pelargonium graveolens*, *Cinnamomum cassia*, *Ocimum basilicum*, and *Eugenia caryophyllus* essential oils. Molecules.

[87] Labib G.S., Aldawsari H. (2015). Innovation of natural essential oil-loaded Orabase for local treatment of oral candidiasis. Drug Des. Devel. Ther..

[88] Hirai A., Odani H., Nakajima A. (1991). Determination of degree of deacetylation of chitosan by ^1^H NMR spectroscopy. Polym. Bull..

[89] Gamzazade A.I., Slimak V.M., Skljar A.M., Stykova E.V., Pavlova S.S.A., Rogozin S.V. (1985). Investigation of the hydrodynamic properties of chitosan solutions. Acta Polym..

[90] Zhao H., Heindel N.D. (1991). Determination of degree of substitution of formyl groups in polyaldehyde dextran by the hydroxylamine hydrochloride method. Pharm. Res..

[91] Moore S., Stein W.H. (1948). Photometric ninhydrin method for use in the chromatography of amino acids. J. Biol. Chem..

[92] Leane M.M., Nankervis R., Smith A., Illum L. (2004). Use of ninhydrin assay to measure the release of chitosan from oral solid dosage forms. Int. J. Pharm..

[93] Xiang C., Zhang X., Zhang J., Chen W., Li X., Wei X., Li P.A. (2022). Porous Hydrogel with High Mechanical Strength and Biocompatibility for Bone Tissue Engineering. J. Funct. Biomater..

[94] Suflet D.M., Popescu I., Pelin I.M., Ichim D.L., Daraba O.M., Constantin M., Fundueanu G. (2021). Dual Cross-Linked Chitosan/PVA Hydrogels Containing Silver Nanoparticles with Antimicrobial Properties. Pharmaceutics.

[95] Gulcin I., Elmastas M., Aboul-Enein H.Y. (2012). Antioxidant activity of clove oil—A powerful antioxidant source. Arab. J. Chem..

[96] CLSI (2012). Methods for Dilution Antimicrobial Susceptibility Tests for Bacteria that Grow Aerobically, Approved Standard.

[97] Lambert R.J.W., Pearson J. (2000). Susceptibility testing: Accurate and reproducible minimum inhibitory concentration (MIC) and non-inhibitory concentration (NIC) values. J. Appl. Microbiol..

[98] Bauer A.W., Kirby W.M.M., Sherris J.C., Turck M. (1966). Antibiotic sensitivity testing by a standardized single disk method. Am. J. Clin. Path..

[99] CLSI (2010). Methods for Antimicrobial Dilution and Disk Susceptibility of Infrequently Isolated or Fastidious Bacteria, Approved Guideline.

