# Supplementary material for: Chitosan–Oxidized Pullulan Hydrogels Loaded with Essential Clove Oil: Synthesis, Characterization, Antioxidant and Antimicrobial Properties
Source: Gels. 2024 Mar 26;10(4):227. doi: 10.3390/gels10040227 (PMC11049474; doi:10.3390/gels10040227)
Supplement: Supplementary file 1 [file gels-10-00227-s001.zip › gels-2927398-supplementary.pdf]

Electronic Supplementary Information (ESI)

Chitosan–Oxidized Pullulan Hydrogels Loaded with Essential Clove Oil: Synthesis, Characterization, Antioxidant and Antimicrobial Properties

Dana Mihaela Suflet [1], Marieta Constantin [1,*], Irina Mihaela Pelin [1], Irina Popescu [1], Cristina M. Rimbu [2],
Cristina Elena Horhogea [2] and Gheorghe Fundueanu [1]

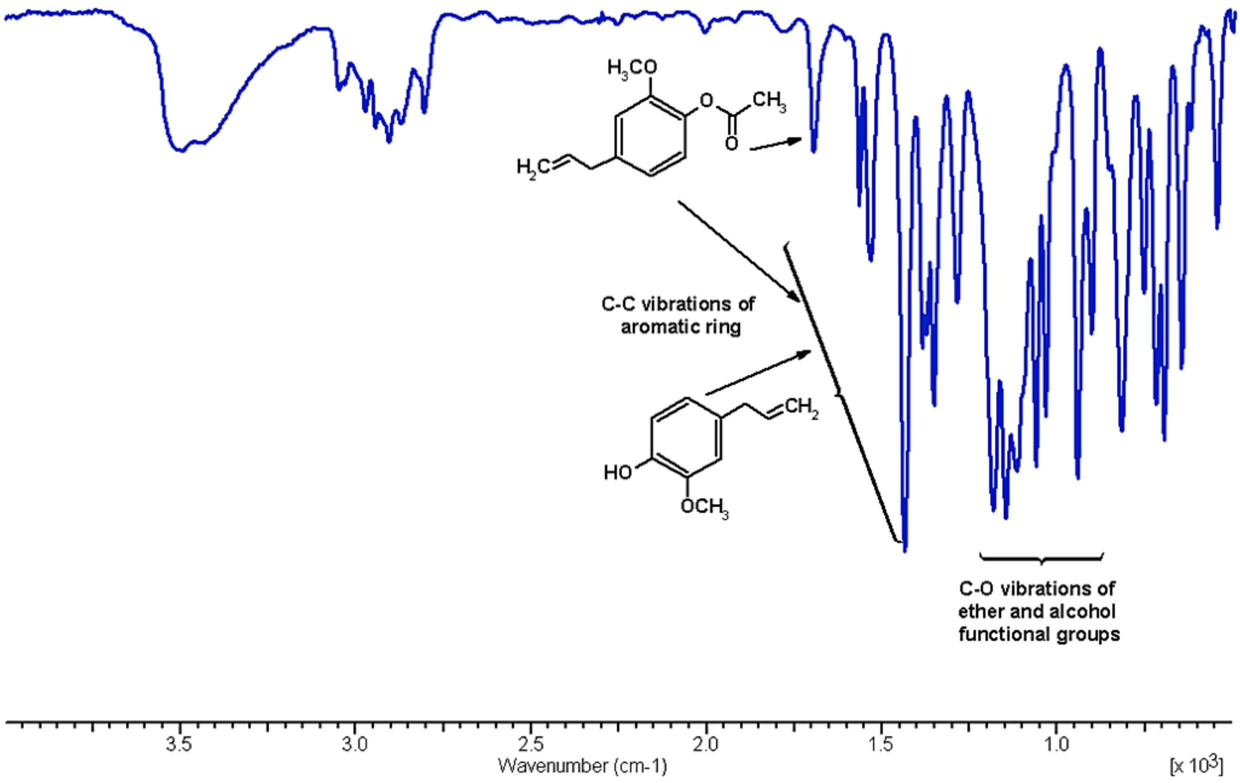

Figure S1. FT-IR spectrum of Clove oil.

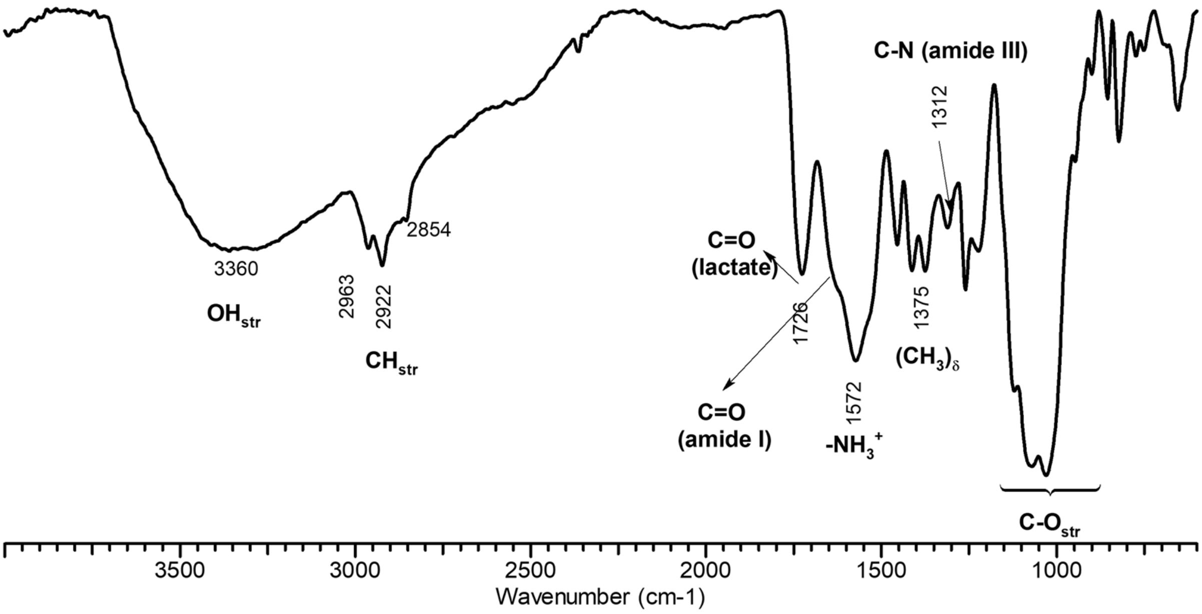

Figure S2. FT-IR spectrum of Chitosan lactate.

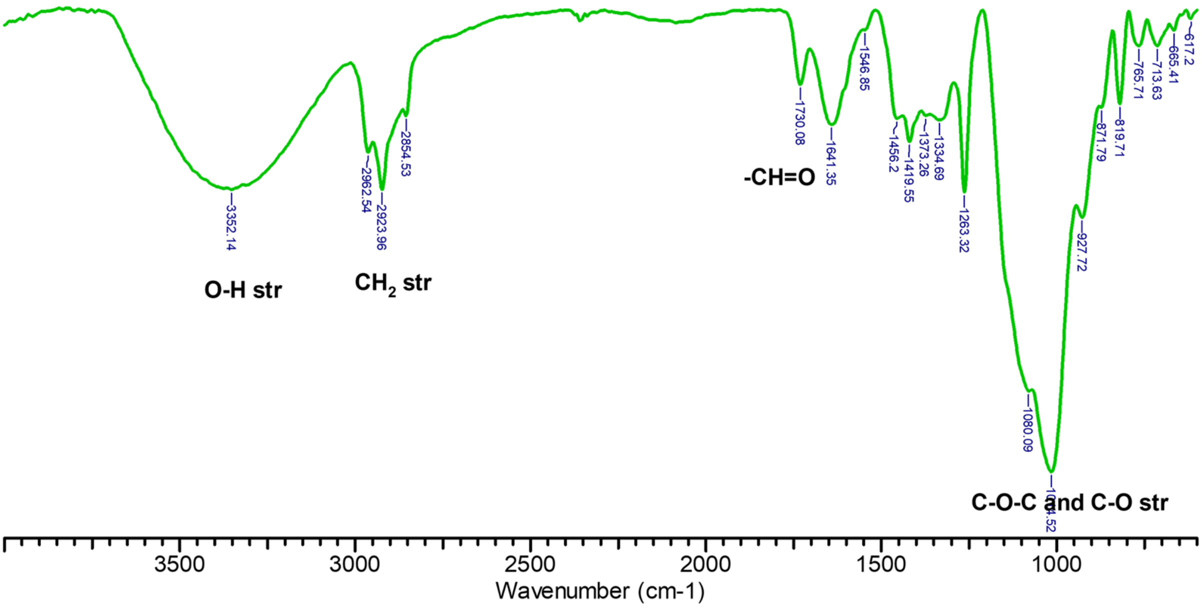

Figure S3. FT-IR spectrum of oxidized pullulan (OP).

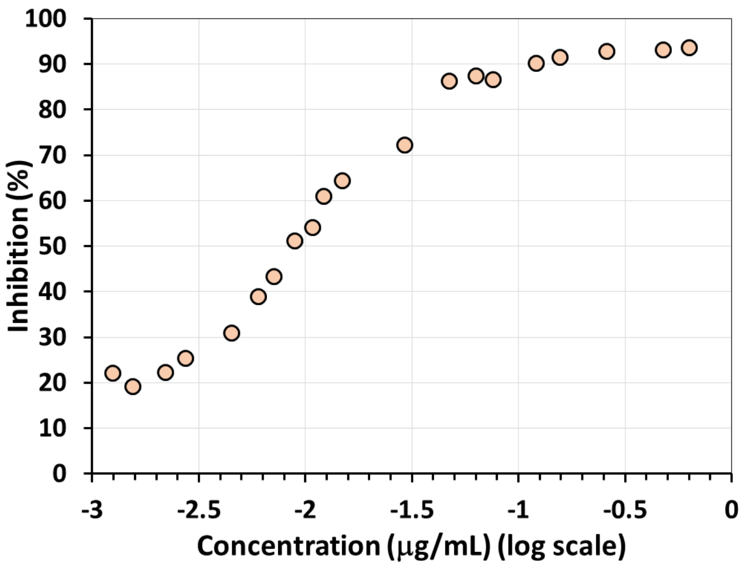

Figure S4. DPPH radical scavenging capacity of Clove oil.

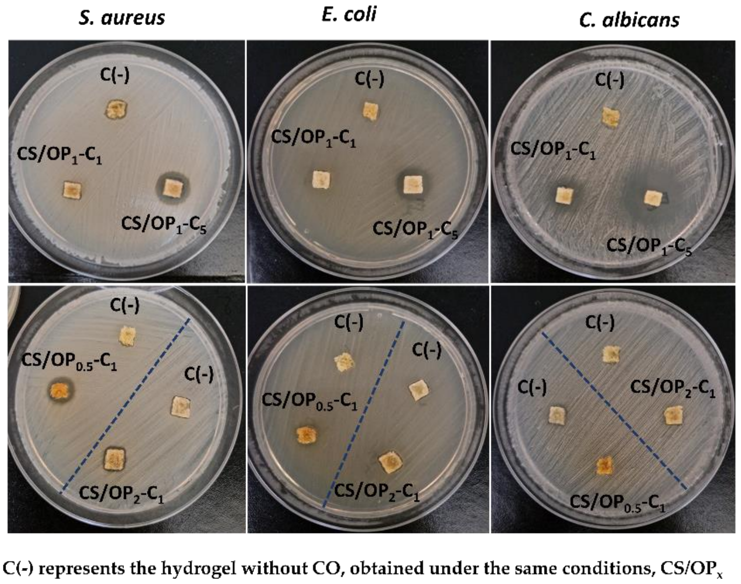

Figure S5. Antibacterial activity of un-loaded and CO-loaded CS/OP hydrogels against *S. aureus, E. coli,* and *C. albicans* through well diffusion assay.
